# Early Depth Engagement in Art Perception: visual dynamics and aesthetic experience

**DOI:** 10.3389/fpsyg.2026.1781822

**Published:** 2026-04-16

**Authors:** Yuka Nojo

**Affiliations:** Graduate School of Arts and Sciences, The University of Tokyo, Meguro-ku, Tokyo, Japan

**Keywords:** depth perception, EDEN, EMHMM, gaze pattern, *Ukiyo-e*

## Abstract

**Background:**

Understanding how aesthetic experience develops over time during art viewing has become an important issue in recent research. However, it remains unclear which perceptual factors engaged during viewing contribute to subsequent gaze organization and aesthetic evaluation.

**Objective:**

This study examined whether gaze engagement with compositional depth is associated with later differences in gaze dynamics and aesthetic evaluation. This temporal pattern is defined here as Early Depth Engagement in Art Perception (EDEN).

**Methods:**

Forty-eight participants freely viewed twelve Japanese *ukiyo-e* landscape prints by Hokusai and Hiroshige for 30 s while their eye movements were recorded. Depth points were defined using a separate depth localization task with the same participants. Gaze metrics were analysed in 3-s windows, and aesthetic ratings were normalized within participants to classify artworks into high- and low-evaluation groups. The same analyses were also applied to a secondary stimulus set of twenty landscape prints by contemporaneous *ukiyo-e* artists.

**Results:**

No group differences were observed during the initial viewing phase (0–6 s), when gaze behavior was exploratory in both groups. From approximately 6 s onwards, the high-evaluation group showed greater fixation frequency and dwell time at depth points, and these differences were maintained over time. In contrast, no consistent group differences were found in time to first fixation on depth points. A similar but weaker temporal divergence was observed in the secondary stimulus set. These findings indicate that compositional depth points function as structural anchors for gaze during the mid-viewing phase rather than as targets of earlier initial access.

**Conclusion:**

EDEN describes not immediate access to depth itself, but the temporal emergence, divergence, and maintenance of depth-related gaze organization during free viewing. This framework provides a dynamic account of how depth-related information becomes incorporated into viewing behavior and contributes to aesthetic experience.

## 1 Introduction

Depth in pictorial space has traditionally been discussed in terms of realism and the accuracy of spatial representation (Arnheim, 1974; Wardle and Watt, 2017). In the context of aesthetic appreciation, however, depth may play a broader role, contributing not only to spatial depiction but also to subjective evaluation and the overall viewing experience (Carbon, 2017; Leder et al., 2004). Although depth cues are known to influence how viewers interpret pictorial space, the relationship between depth perception and aesthetic evaluation remains insufficiently understood, particularly with respect to how such information is accessed and organized over time.

Previous research has shown that aesthetic experience unfolds dynamically rather than being determined at a single moment (Locher et al., 2008; Smith and Smith, 2001). Nevertheless, empirical studies have typically examined depth perception using simplified or artificial stimuli, static compositional analyses, or explicit judgment tasks. As a result, little is known about how viewers engage with depth-related information during naturalistic, free viewing of artworks, or how such engagement is reflected in the temporal organization of gaze behavior. In particular, it remains unclear how engagement with compositional depth cues develops during the viewing process, and whether temporal differences in gaze behavior are associated with differences in aesthetic evaluation.

From a theoretical perspective, depth perception is supported by interacting perceptual processes operating at early and intermediate stages of visual processing. Low- and mid-level cues such as occlusion, perspective, and shading enable observers to construct meaningful spatial structure from two-dimensional input, even when metric reconstruction is ambiguous or incomplete (Marr, 1982; Adelson, 2000). Contemporary accounts of perception further emphasize that such spatial organization emerges progressively through interactions between sensory input and internal models (Friston, 2010). In models of aesthetic experience, early perceptual analyses are regarded as foundational for later evaluative processes (Leder et al., 2004). Despite these converging theoretical perspectives, quantitative evidence linking the temporal dynamics of depth-related perception to aesthetic experience—particularly through eye-movement analysis—remains limited (Francuz et al., 2018).

To address this gap, the present study introduces Early Depth Engagement in Art Perception (EDEN) as a descriptive framework for characterizing how engagement with compositional depth cues is organized over time during free viewing. EDEN does not posit a specific cognitive mechanism, nor does it assume an immediate or causal influence of depth cues on aesthetic judgment. Rather, it provides a temporal viewpoint for organizing empirically observable gaze patterns, allowing examination of when and how depth-related information is incorporated into viewing behavior without presupposing its evaluative role.

The study employs a two-stage experimental design consisting of a primary and a secondary stimulus set. The primary stimulus set comprises twelve landscape prints by Katsushika Hokusai and Utagawa Hiroshige, previously used in related work (Nojo and Chan, 2025). *Ukiyo-e* landscapes from this period were not based on a single, systematically applied perspective system; instead, spatial depth is often suggested through heterogeneous and sometimes ambiguous compositional cues. Such characteristics provide a suitable context for examining differences in perceptual access to depth cues during free viewing. The secondary stimulus set is composed of 20 landscape prints by contemporaneous *Ukiyo-e* artists, selected to examine whether similar patterns of engagement with depth cues can be observed beyond a limited set of canonical works.

The present study examines how engagement with compositional depth cues unfolds over time during free viewing, using time-resolved gaze analysis and the EDEN framework to describe temporal patterns of viewing behavior across artworks.

## 2 Previous studies

Depth perception is supported not only by higher-level cues such as binocular disparity but also by low- to mid-level visual processing based on monocular cues, including shading, gradients, and orientation structure. Research in visual science has demonstrated that such local visual features are utilized at early stages of visual processing to infer three-dimensional structure from two-dimensional input. For example, (Aubuchon et al. 2025) showed that orientation information derived from shading in natural images can predict human three-dimensional shape perception with high accuracy, suggesting that depth-related information is available early in visual processing (see also Adelson, 2000; Marr, 1982; Todd, 2004). However, this line of research has largely relied on static stimuli, and the temporal organization of depth perception during ongoing viewing has not been systematically examined.

From the perspective of aesthetic and material evaluation, several models have proposed that subjective judgment is supported by multiple perceptual dimensions, among which depth constitutes a core component rather than a secondary factor (e.g., Leder et al., 2004; Reber et al., 2004; Schmidt et al., 2025). While these models establish the importance of depth within evaluative structure, they are primarily concerned with static descriptions of perceptual dimensions and do not address how such dimensions become engaged or organized over time during viewing.

Within art theory and empirical aesthetics, the role of depth representation in shaping viewing experience and impression formation has also been extensively discussed (e.g., Arnheim, 1974; Carbon, 2017; Wardle and Watt, 2017; Wijntjes, 2012). This body of work has provided valuable insights into compositional strategies and perceptual effects of depth in pictorial space. Nevertheless, much of this literature is based on static compositional analysis, offering limited insight into how viewers access and organize depth-related information during the temporal progression of actual viewing behavior.

Taken together, prior research across visual science, evaluation models, and art theory converges on the view that depth is a fundamental perceptual dimension in pictorial experience. At the same time, it remains unclear how engagement with compositional depth cues unfolds over time during free viewing, or how such engagement is reflected in the temporal organization of gaze behavior.

Finally, the present study is theoretically continuous with prior research on RAIC (Reading of an Artist's Intention from the Composition), which examines how viewers infer artistic intention from compositional structure (Nojo and Chan, 2025). From this perspective, depth perception is positioned as a perceptual foundation that precedes RAIC, motivating a temporal-dynamic examination of how such perceptual organization develops during art viewing.

## 3 Materials and methods

### 3.1 Participants

The sample size for the present study was determined based on statistical power considerations and consistency with prior eye-movement research. Given the substantial inter-individual variability typically observed in gaze behavior, we assumed a medium effect size (*r* = 0.30; Cohen, 1988), corresponding to Cohen's *d* = 0.628. Under this assumption, a one-tailed test with α = 0.05 and statistical power of 0.80 requires approximately 16 participants per group. Because participants were divided into three evaluation-based groups (high, medium, and low), a total sample size of 48 participants was selected. This sample size is consistent with previous studies employing gaze pattern analysis in art perception (Chuk et al., 2014) and with our prior work using similar analytical frameworks (Nojo and Chan, 2025).

For the experiment using the primary stimulus set, 48 undergraduate and graduate students (30 males, 18 females; mean age = 22.2 years, SD = 3.37) participated. The participant composition and artwork-specific exclusion criteria for this stimulus set were identical to those reported in the previous RAIC study (Nojo and Chan, 2025), allowing for methodological continuity and comparability across studies. In the present analyses, participant data judged to be valid for each artwork were treated as the unit of analysis.

For the experiment using the secondary stimulus set, an initial sample of 48 undergraduate and graduate students (23 males, 25 females; mean age = 21.8 years, SD = 1.81) participated. Eye-movement data from three participants were not recorded due to technical problems with the experimental videos or the eye-tracking equipment. These participants were excluded, and data from the remaining 45 participants were included in the analyses (see Table 1).

**Table 1:** Number of participants and analyzed samples for each stimulus set.

Stimulus	No. of participants	No. of images	Samples	Excluded conditions
Primary stimulus set	45–48	12	6,780	Trails in which fixation data were missing continuously for more than 2 s.
Secondary stimulus set	45	20	18,000	Data loss caused by malfunction of the eye-tracking device or the video camera.

The table summarizes the number of participants, images, and valid samples included in the analyses for the primary and secondary stimulus sets. For the primary stimulus set, the number of participants varied across images due to image-specific exclusion criteria, and analyses were conducted using only valid participant data for each artwork. For the secondary stimulus set, participants with missing or invalid eye-tracking data were excluded prior to analysis.

All participants had normal or corrected-to-normal vision, reported no history of neurological or visual disorders, and had not received formal or professional training in art. Written informed consent was obtained prior to participation. The study was approved by the ethics committee of the university with which the first author is affiliated, and all procedures were conducted in accordance with the relevant ethical guidelines and regulations. Participants received monetary compensation after completing the experiment.

### 3.2 Apparatus and recording setup

Eye movements were recorded using a Tobii TX300 Pro eye-tracking system (Tobii Technology, Stockholm, Sweden) with a sampling rate of 300 Hz. Stimuli were presented on a 23-inch monitor with a resolution of 1920 × 1080 pixels. Participants were seated at a viewing distance of approximately 55 cm, resulting in a visual angle of approximately 50 ° × 29 °. A five-point monocular calibration procedure was performed separately for each eye prior to the experiment. Calibration accuracy was visually inspected and repeated if necessary. Head movements were not restricted, but participants were instructed to maintain a stable viewing posture throughout the experiment. Stimuli were presented in full-screen mode against a black background. The order of stimulus presentation was randomized across participants.

### 3.3 Stimuli

**Figure 1:**
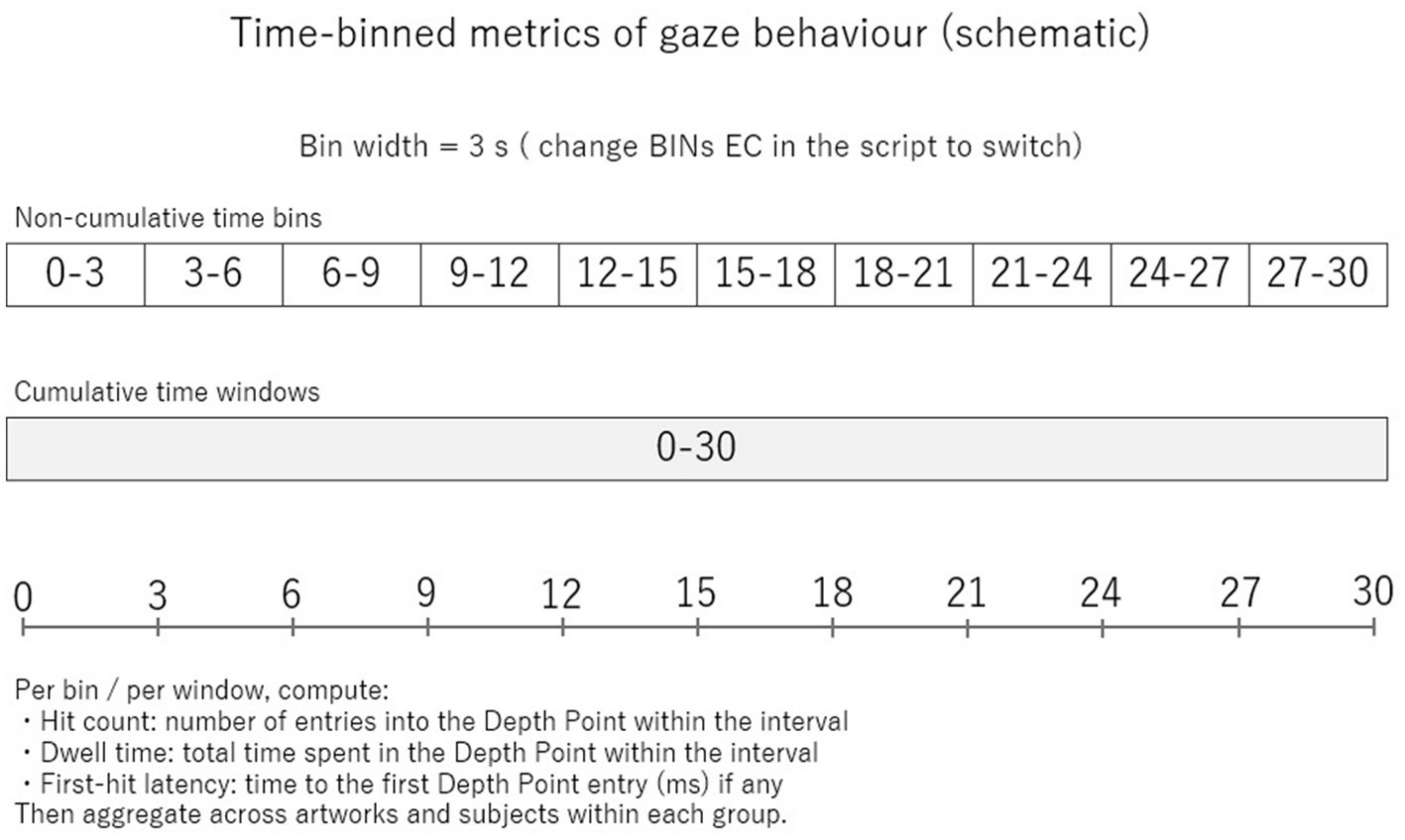
Schematic overview of time-binned gaze analysis. Schematic illustration of the time-binning procedure used to analyze gaze behavior during free viewing. Eye-movement data recorded over the 30-s viewing period were segmented into non-cumulative time bins of 3 s (0–3, 3–6, …, 27–30 s) as well as cumulative time windows spanning the entire viewing interval (0–30 s). For each time bin or window, multiple gaze metrics were computed, including hit count (number of entries into the Depth Point within the interval), dwell time (total fixation duration within the Depth Point), and first-hit latency (time to the first entry into the Depth Point, when applicable). These metrics were analyzed separately for non-cumulative and cumulative time windows and subsequently aggregated across artworks and participants within each aesthetic-evaluation group.

Japanese *Ukiyo-e* landscape prints were used as visual stimuli in this study. The stimuli consisted of two sets. The primary stimulus set comprised twelve landscape prints by Katsushika Hokusai and Utagawa Hiroshige, which were identical to those used in a previous study (Nojo and Chan, 2025). *Ukiyo-e* landscapes of this period did not employ a single, systematically applied perspectival framework; instead, spatial depth was suggested through a variety of compositional cues that were heterogeneous and, in some cases, perceptually ambiguous. These characteristics provide particularly suitable conditions for examining how differences in perceptual access to depth-related cues during free viewing are associated with variations in gaze behavior and aesthetic evaluation. In particular, the diverse depth cues inherent in landscape prints naturally elicit exploratory gaze behavior, thereby offering an ecologically valid context for investigating how viewers access depth information and how such information becomes organized during viewing. The secondary stimulus set consisted of 20 landscape prints by *Ukiyo-e* artists who were active during the same period as Hokusai and Hiroshige. These works were primarily selected from prints produced in the early nineteenth century (approximately 1818–1844). The selected artworks included compositional elements suggestive of spatial depth, such as rivers, ferry crossings, highways, shrines and temples, and distant mountains. To avoid bias in viewing content or thematic focus, artworks in which human figures constituted the primary subject were excluded, and only stimuli centered on landscape representation were used. All stimulus images were high-resolution digital reproductions obtained from museum collections in Japan and abroad. During the experiment, each stimulus was presented individually as a single image on the display. The titles of the artworks and the number of participants assigned to each evaluation group are summarized in Supplementary Table 1. A detailed list of the stimulus artworks is provided in the Supplementary Figure 1. The primary stimuli are identical to those presented in Figure 1 of (Nojo and Chan 2025), reproduced here for completeness.

### 3.4 Procedure and experimental tasks

For each stimulus set, two experimental tasks were conducted with the same participants: an Impression Rating Task and a Depth Localization Task. The tasks were performed independently, and participants completed each task according to task-specific instructions. The order of tasks was counterbalanced across participants. The experimental procedures and part of the data for the primary stimulus set—specifically those related to the Impression Rating Task—have been reported previously (Nojo et al., 2023; Nojo and Chan, 2025). The present study extends these procedures by integrating a depth localization task and by focusing on the temporal dynamics of gaze behavior during free viewing.

#### 3.4.1 Impression rating task

Each trial began with the presentation of a central fixation cross on a white background for 5 s. A stimulus image was then randomly displayed for 30 s, during which participants were instructed to view the artwork freely. Following stimulus presentation, participants rated the artwork on four dimensions—beauty, liking, quality of composition, and quality of color—using a 10-point scale controlled by mouse input. Participants also indicated whether they had previously seen the artwork using a binary (yes/no) response. In addition to numerical ratings, participants were asked to verbally describe their overall impression of the artwork. These verbal reports were recorded with participants' consent and were used as Supplementary information to aid interpretation of the evaluative judgments.

#### 3.4.2 Depth localization task

In the depth localization task, participants first completed a practice trial using a sample image. In each experimental trial, a rest image was presented for 5 s prior to stimulus onset, followed by presentation of a stimulus image. Participants were instructed to use a handheld pointer to indicate the location within the artwork that they perceived as exhibiting the greatest depth. After indicating the location, participants verbally described the selected area and reported the perceived distance to that location. This distance report did not correspond to a physical or metric distance but reflected the magnitude of depth as subjectively perceived by the participant and was treated as a relative measure. No time constraints were imposed on either the pointing response or the verbal report. The on-screen position of the pointer and the entire sequence of verbal responses were recorded using a video camera with participants' consent. These recordings were collected as Supplementary material to support interpretation of perceptual and evaluative responses.

## 4 Data analysis

The analyses were designed to characterize the temporal organization of gaze behavior during free viewing and to examine how engagement with compositional depth cues unfolds over time in relation to aesthetic evaluation. Rather than testing effects at isolated time points, the analyses focused on how gaze patterns diverge, persist, and stabilize across the viewing period. The analytical approach adopted in this study is descriptive in nature, and the gaze measures were interpreted jointly as complementary descriptors capturing different aspects of gaze behavior and its temporal organization during viewing. An overview of the analysis pipeline, illustrating the preprocessing steps, parallel analytical streams, and derived metrics, is provided in Supplementary Figure 2.

Eye movements were recorded continuously during the 30 s presentation of each stimulus at a sampling rate of 300 Hz. The viewing period was segmented into consecutive 3 s time windows, allowing for time-resolved analyses across the full viewing duration. This temporal resolution was chosen to subdivide the commonly cited initial exploratory phase of approximately 5–6 s into finer phases (e.g., Locher et al., 2008; Smith and Smith, 2001), enabling differentiation between early exploratory behavior (0–3 s) and early stabilization processes (3–6 s), while avoiding assumptions of temporally uniform effects across viewing.

Subjective evaluation scores exhibit substantial inter-individual variability. To account for this, all evaluation values were normalized within participants prior to analysis. To preserve artwork-specific evaluative structure, normalization and subsequent group classification were performed independently for the Primary stimulus set and the Secondary stimulus set. For each artwork, participants were classified into high- and low-evaluation groups based on normalized aesthetic ratings, and group comparisons were conducted at the artwork level.

Analyses were conducted at multiple, complementary levels. First, fixation-based measures were computed for independently defined depth points obtained from the Depth Localization Task. These measures included fixation count, cumulative dwell time, and revisit behavior, capturing the degree to which viewers selectively and persistently allocated overt attention to depth-related regions of the composition.

Second, to characterize the temporal structure of gaze behavior more comprehensively, Eye Movement Hidden Markov Models (EMHMM) were applied to represent continuous gaze trajectories as sequences of discrete gaze states and their transitions. From these models, state distribution measures such as normalized entropy (Hnorm) were derived for each time window, quantifying the dispersion and concentration of gaze behavior over time. Lower entropy values indicate more concentrated and organized gaze patterns, whereas higher values reflect more distributed and exploratory viewing. The gaze states estimated by EMHMM were derived inductively from the data and represent spatial configurations of fixation behavior without presupposing predefined perceptual units or cognitive mechanisms.

In addition, explicit depth localization measures, including localization time and subjective distance reports obtained from the Depth Localization Task, were analyzed to characterize how participants accessed depth-related information under explicit task conditions. These measures were not interpreted as direct indicators of aesthetic judgment formation but were used as an independent reference for relating free-viewing gaze behavior to explicit depth perception. Group comparisons were conducted using non-parametric statistical tests appropriate to the distributional properties of the data. In addition to *p* values, effect sizes were reported to facilitate interpretation of the magnitude of observed differences.

### 4.1 Depth points

Depth points for each stimulus set were derived from the results of the Depth Localization Task. Specifically, the locations indicated by participants as the point of greatest perceived depth using a handheld pointer were collected, and between one and six depth points were defined for each artwork. Next, gaze heatmaps were generated from the eye-movement data and linked to the corresponding depth points, which were then defined in terms of their spatial coordinates. In addition, the experimental videos were used to record, in seconds, (a) the measurement start time, (b) the time point at which the participant indicated a depth point with the pointer, and (c) the time point at which the participant completed the verbal distance report. Depth points for each artwork were defined based on the spatial distribution of locations that participants judged as having the greatest depth in the depth perception task. To this end, each stimulus image was divided into a uniform grid of 32 × 16 cells, and the grid cell containing each participant's indicated depth location was recorded. The number of indications for each cell was then aggregated across participants, yielding a spatial frequency distribution (heatmap) of depth judgments for each artwork. Although this distribution reflects individual variability, it was interpreted as capturing the central regions of depth perception shared across viewers for a given artwork. Depth point regions of interest (ROIs) were extracted by identifying contiguous groups of grid cells that exceeded a predefined threshold of participant responses within this frequency distribution. Each depth point ROI was then defined by its minimum bounding rectangle (minX, maxX, minY, maxY). Through this procedure, depth points were represented not as single spatial locations, but as spatially extended regions corresponding to areas of depth perception commonly identified by multiple participants.

#### 4.1.1 Two-group comparisons at depth points

Based on the results of the Depth Localization Task, we examined whether significant differences were present between the high- and low-evaluation groups (defined on an artwork-by-artwork basis) for the following three depth-related measures:

(1) the perceived magnitude of depth (Distance_km),
(2) the time required to recognize the depth point (Depth recognition completion time), and
(3) the time required to estimate the depth distance (Calculate distance time).

For each artwork, these measures were compared between the two groups to assess whether depth perception and its associated temporal characteristics differed as a function of aesthetic evaluation.

### 4.2 ROI extraction using EMHMM

Regions of interest (ROIs) based on gaze data were extracted using the Eye Movement Hidden Markov Model (EMHMM; Chuk et al., 2014; Hsiao et al., 2021). EMHMM is a probabilistic modeling framework that simultaneously estimates spatial fixation distributions and their temporal transition structure from eye-movement sequences of multiple observers, allowing representative gaze states (ROIs) to be identified in a data-driven manner. In the present study, for each stimulus image, participants' gaze sequences (x- and y-coordinates) were used as input to estimate fixation states represented by Gaussian distributions, together with the transition probabilities between these states. To avoid confounding effects due to differences in image size, all stimulus images and gaze coordinates were normalized to a common resolution prior to analysis. The number of ROIs was fixed at 14 states. This choice was based on preliminary analyses showing that increases in model likelihood saturated asymptotically as the number of states increased, such that further subdivision did not yield additional interpretative benefits. This setting is also consistent with prior studies in which a comparable number of states has been shown to provide a stable representation of gaze patterns. To reduce the influence of dependence on initial parameter values, model training was performed with multiple random initializations, and the solution yielding the highest log-likelihood was selected. To prevent the emergence of excessively large or small ROIs, statistical constraints were applied to the area and axis lengths of the ROIs estimated by EMHMM. Specifically, ROI areas were restricted to fall within 0.1%−5% of the total image area. In addition, the lengths of the ROI axes were constrained using a clamping procedure based on the mean ± N standard deviations of the ROI distribution, ensuring that individual ROIs did not deviate excessively from the overall distribution. Each learned ROI was represented as a two-dimensional Gaussian distribution defined by its mean location and covariance, thereby characterizing both the central position and spatial extent of each fixation region.

#### 4.2.1 Procedure for computing gaze-state sequence metrics based on HMM

##### 4.2.1.1 State series inference from gaze data

First, the ROIs (K = 14) learned by EMHMM in the preceding step were treated as the observation space, and gaze time-series data were transformed into hidden state sequences for each participant and each artwork. Specifically, the observation sequence consisted of gaze coordinates at each time point, while the hidden states corresponded to the ROIs defined by EMHMM. Using the trained HMM parameters, both the posterior state probabilities for each state at each time point and the most likely state sequence (Viterbi path) were estimated. Through this procedure, continuous gaze trajectories were represented as temporal sequences of discrete gaze states, enabling subsequent analyses of gaze dynamics at the level of state transitions and dwell patterns.

##### 4.2.1.2 Aggregation of state distributions across temporal windows

The inferred gaze-state sequences were aggregated into successive temporal windows of fixed duration (3 s), covering the entire 30-s viewing period from stimulus onset. Within each temporal window, the following three measures were computed:

(1) the probability distribution over states, obtained by averaging posterior state probabilities within the window;
(2) the number of visits to each state (hit count); and
(3) the total dwell time for each state.

Through this procedure, each temporal window was quantitatively characterized as a distribution of gaze states representing the viewer's gaze behavior during that specific time interval.

##### 4.2.1.3 Uncertainty and concentration measures of gaze distributions

(a) **State Entropy**

To quantify the uncertainty of the gaze-state distribution within each temporal window, Shannon entropy was computed. Entropy provides a measure of how broadly or narrowly gaze is distributed across states, with higher values indicating a more dispersed and exploratory distribution of gaze states, and lower values indicating greater concentration on a limited subset of states.


$$H=-\overset{K}{\underset{i=1}{\sum }}p_{i}\log p_{i}$$

Here, *p*_*i*_ denotes the mean posterior probability of state *i* within a given temporal window. Higher entropy values indicate that gaze is distributed across multiple states, reflecting a more exploratory viewing pattern, whereas lower values indicate greater concentration on a specific subset of states. To facilitate comparability across analyses, entropy values were normalized by the theoretical maximum log*K*, yielding a normalized entropy measure (*H*_norm_).

(b) **Maximum State Probability (MaxP)**

From the same state distribution, the probability of the most likely state was computed.


$$\text{MaxP}=\underset{i}{\max}p_{i}\;$$

The maximum state probability (MaxP) represents the strength of the dominant gaze state within a given temporal window and reflects the degree of gaze localization and stabilization during that interval.

(c) **Effective Number of States (Neff)**

To evaluate how many gaze states were effectively engaged within a given temporal window, the effective number of states was computed based on entropy. This measure captures the extent to which gaze is distributed across states in practice, providing an interpretable index of the diversity of gaze allocation beyond the nominal number of states.


$$N_{\text{eff}}=\exp\left(H\right)\;$$

In addition, a normalized measure of the effective number of states was used by dividing by the total number of ROIs, yielding the normalized effective number of states (NeffNorm).

##### 4.2.1.4 Dynamic characteristics of gaze transitions

To capture the temporal dynamics of gaze-state sequences, the following transition-based measures were computed.

(d) **Total Variation(TV)**

The difference between state distributions across successive temporal windows was quantified as a distance between probability distributions, providing a measure of the magnitude of change in gaze-state distributions over time.

(e) **Switch Rate**

The frequency of state switches in the most likely state sequence (Viterbi path) was computed, reflecting the degree of exploratory switching in gaze behavior.

#### 4.2.2 Aggregation and statistical analysis for group comparisons

All metrics were computed for each artwork and each temporal window and then compared between the high- and low-evaluation groups classified based on aesthetic ratings. Group comparisons were conducted using non-parametric statistical tests, selected according to the distributional properties of the data. For each comparison, *p*-values and effect sizes (rank-biserial correlation) were reported.

## 5 Result

### 5.1 Emergence of temporal divergence after 6 s

In this section, we analyzed the temporal evolution of gaze behavior during 30 s of free viewing for twelve landscape prints by Hokusai and Hiroshige (primary stimulus set, *n* = 48) and examined when and how gaze behavior diverged between the high- and low-evaluation groups. Because the unit of analysis varied depending on the specific measure, the n values reported below do not necessarily correspond to the number of participants.

**Figure 2:**
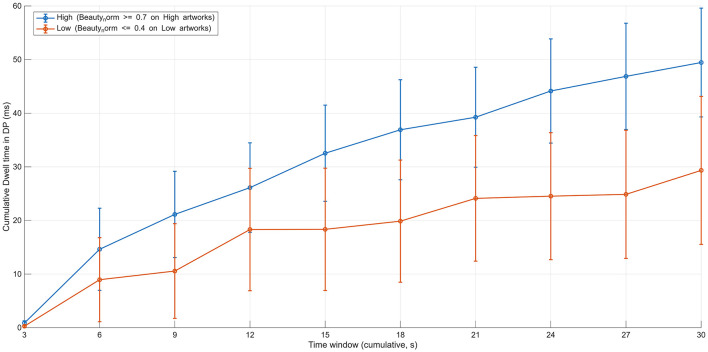
Time-course of cumulative dwell ratio at depth-related points (DP) during free viewing (12 images). The figure shows the cumulative proportion of fixation time allocated to depth-related points (DP) as a function of viewing time (3–30 s) for the high- and low-evaluation groups. During the early viewing phase (0–6 s), both groups exhibited similarly low DP dwell ratios with substantial variability. After approximately 6 s, the high-evaluation group consistently showed higher cumulative DP dwell ratios than the low-evaluation group, and this difference was maintained until the end of the viewing period. Error bars indicate ±1 SD.

During the initial exploratory phase (0–6 s), no stable group differences were observed between the high- and low-evaluation groups for any of the major gaze metrics related to depth points (DPs), including fixation frequency, cumulative dwell-time ratio, revisit count, or mean dwell time per visit. For example, the cumulative DP dwell-time ratio remained very low for both groups at 3 s (high-evaluation group ≈0.3 × 10^−3^, low-evaluation group ≈0.1 × 10^−3^), and both groups remained at a comparable order of magnitude at 6 s (high-evaluation group ≈2.4 × 10^−3^, low-evaluation group ≈1.5 × 10^−3^), with substantial variability (Figure 2). At this stage, fixations on depth points were observed only in a subset of participants, and gaze behavior in both groups was dominated by dispersed, exploratory viewing of the overall composition.

**Figure 3:**
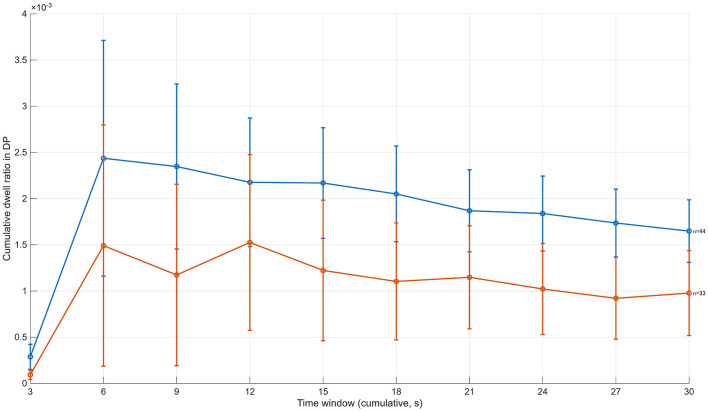
Time-course of cumulative revisits to depth-related points (DP) during free viewing (12 images). This figure plots the cumulative number of revisits to DP across successive cumulative time windows. While revisit counts were comparable between groups in the initial viewing phase, the high-evaluation group began to show a greater accumulation of revisits after approximately 6 s. This divergence increased gradually over time, indicating sustained re-engagement with depth-related regions in the high-evaluation group. Error bars represent ±1 SD.

By contrast, a clear divergence in gaze behavior began to emerge after approximately 6 s from viewing onset. From the 6–9 s time window onward, the cumulative DP dwell-time ratio was consistently higher in the high-evaluation group than in the low-evaluation group. Specifically, at 9 s, the high-evaluation group showed a value of approximately 2.3 × 10^−3^, compared with approximately 1.2 × 10^−3^ in the low-evaluation group. This difference was maintained in subsequent time windows, including at 12 s (high ≈ 2.2 × 10^−3^, low ≈ 1.5 × 10^−3^) and 15 s (high ≈2.1 × 10^−3^, low ≈1.2 × 10^−3^). Thereafter, the group difference persisted without disappearing up to the end of the viewing period, with values around 1.6 × 10^−3^ for the high-evaluation group and around 1.0 × 10^−3^ for the low-evaluation group at 30 s (Figure 3).

**Figure 4:**
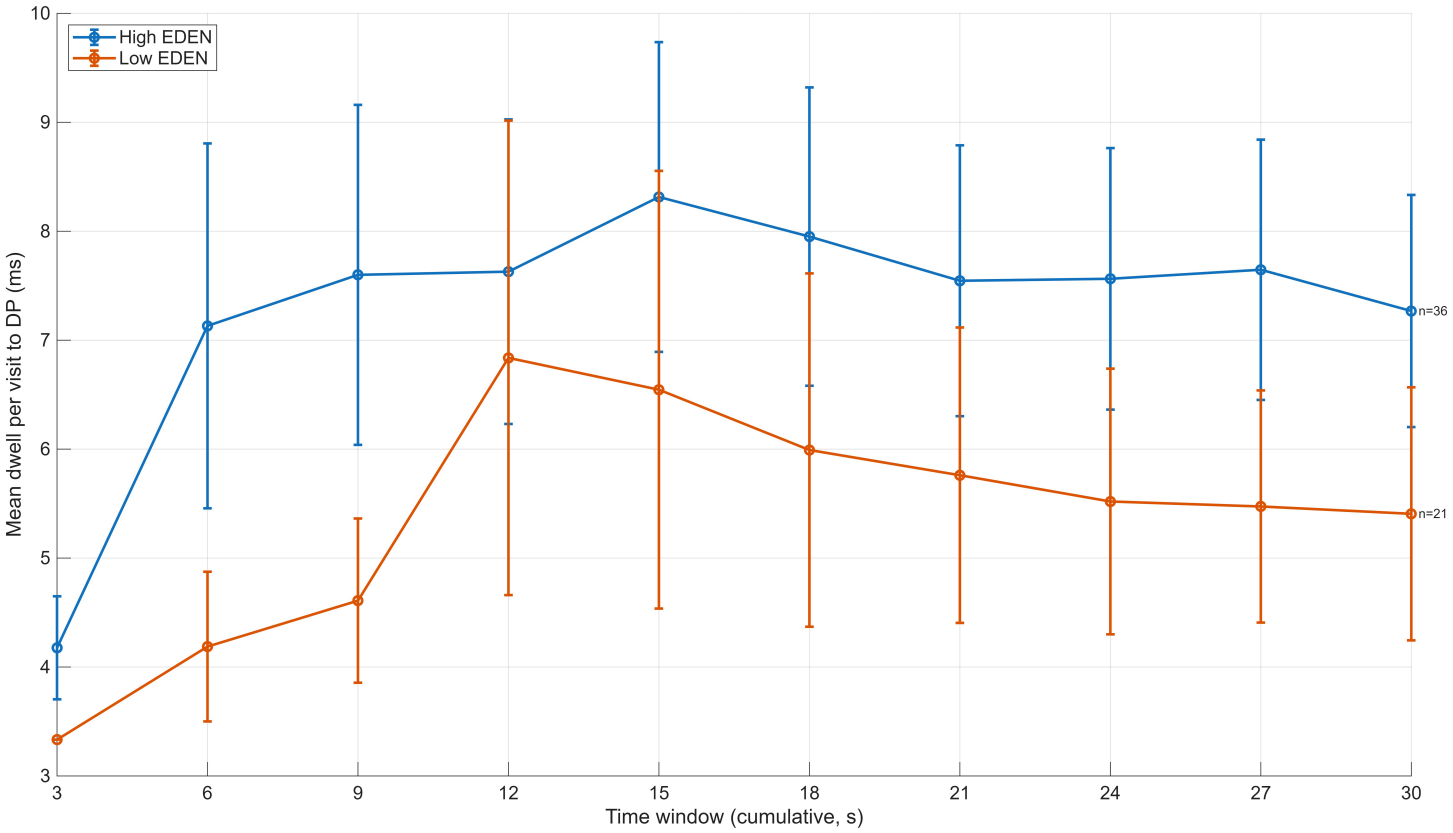
Time-course of mean dwell duration per visit to depth-related points (DP) during free viewing (12 images). The figure depicts changes in the mean dwell duration per visit to DP across cumulative time windows. At around 6 s, the high-evaluation group showed markedly longer dwell durations per visit compared to the low-evaluation group, indicating deeper engagement with DP upon initial access. Although the group difference decreased slightly at later time windows, longer dwell durations in the high-evaluation group were consistently maintained throughout the viewing period. Error bars denote ±1 SD.

A similar temporal divergence was observed for revisit behavior to depth points and for mean dwell time per visit (Figure 4). With respect to mean dwell time, the difference between groups was largest at 6 s, with values of approximately 7.1 ms for the high-evaluation group and 4.2 ms for the low-evaluation group (a difference of about 2.9 ms). At 15 s, mean dwell time was approximately 8.3 ms for the high-evaluation group and 6.5 ms for the low-evaluation group, reducing the difference to about 1.8 ms. In later time windows, mean dwell time stabilized at approximately 7.5–7.7 ms for the high-evaluation group and 5.4–5.6 ms for the low-evaluation group. Although the group difference was smaller than at its initial peak, it was consistently maintained until the end of viewing. These results indicate that the group differences did not increase monotonically over time, but instead emerged prominently during the early phase of divergence and subsequently stabilized at a sustained level.

Considering the temporal profiles of effect magnitude, all observed group differences emerged at approximately 6 s after viewing onset and remained stable across subsequent time windows without disappearing. Thus, the group differences observed after 6 s cannot be attributed to transient fluctuations or effects confined to specific time windows, but rather reflect a persistent divergence characterizing gaze behavior during the mid-viewing phase. This pattern was consistently observed across all twelve artworks and was not driven by any single stimulus.

In contrast, no consistent group differences were observed in the time to first fixation on depth points across any time window. That is, the group differences were not characterized by how quickly participants reached depth points, but by how selectively and persistently they attended to these regions after initial access. Taken together, these findings demonstrate that gaze behavior during free viewing is not temporally homogeneous. Instead, a divergence between the high- and low-evaluation groups emerges at approximately 6 s after viewing onset and is maintained throughout the mid-viewing phase of viewing. As shown in subsequent analyses, this sustained divergence is closely associated with the stabilization and structuring of gaze behavior over time.

### 5.2 Amplification and stabilization of gaze patterns

As shown in Section 4.1, no pronounced group differences were observed during the initial exploratory phase (0–6 s), whereas a temporal divergence in gaze behavior emerged after approximately 6 s. In this section, we examine whether this divergence represents a transient change or whether it persists and accumulates during the mid-viewing phase, manifesting as stabilization of gaze patterns.

First, cumulative dwell time directed to ROIs containing compositional depth points (DPs) was compared between groups. In the high-evaluation group, dwell time around DPs increased consistently after 6 s and was maintained at a relatively high level over time. Specifically, the cumulative DP dwell-time ratio was approximately 2.4 × 10^−3^ at 6 s, remained around 2.1 × 10^−3^ at 15 s, and was still approximately 1.6 × 10^−3^ at 30 s. In contrast, in the low-evaluation group, this measure remained lower, with values of approximately 1.5 × 10^−3^ at 6 s, 1.2 × 10^−3^ at 15 s, and 1.0 × 10^−3^ at 30 s (Figure 2). These results indicate that, during the mid-viewing phase, gaze in the high-evaluation group became increasingly and cumulatively localized around specific compositional elements.

**Figure 5:**
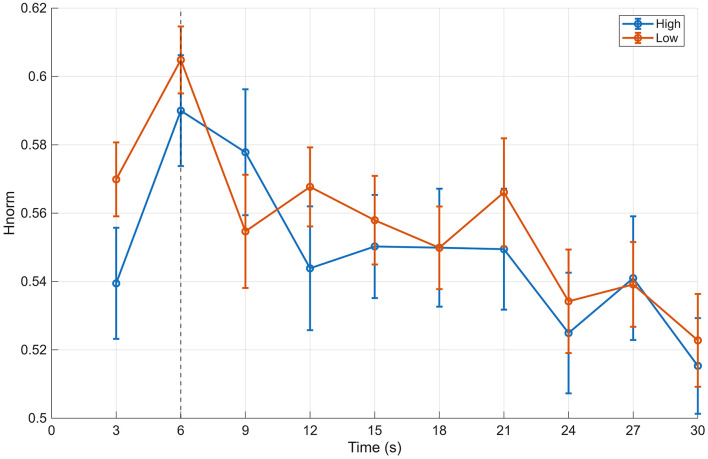
Time-course of Hnorm (BIN) for the 12-image and 20-image stimulus sets. Time-course of normalized entropy (Hnorm) of gaze state distributions, computed in 3-s bins, for high- and low-aesthetic-evaluation groups. Results are shown separately for the 12-image primary stimulus set and the 20-image secondary stimulus set. Points indicate group medians, and error bars represent interquartile ranges. The vertical dashed line denotes the end of the initial viewing phase (6 s).

**Figure 6:**
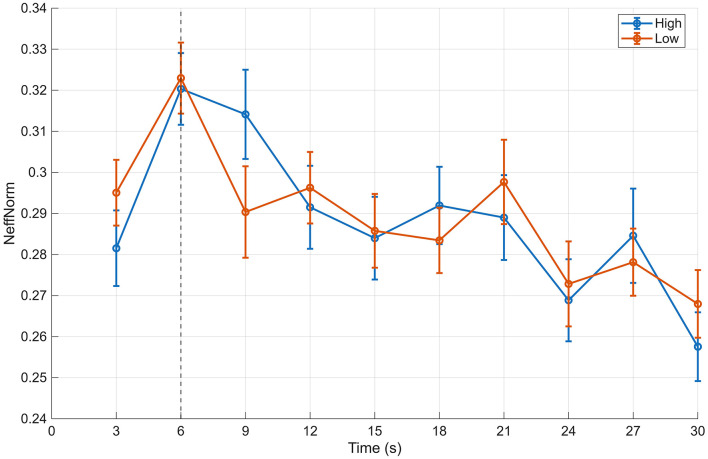
Time-course of NeffNorm (BIN) for the 12-image and 20-image stimulus sets. Time-course of the normalized effective number of gaze states (NeffNorm), computed in 3-s bins, for high- and low-aesthetic-evaluation groups. Data are shown for both the 12-image primary stimulus set and the 20-image secondary stimulus set. Points represent group medians with interquartile ranges. The vertical dashed line indicates the boundary between the initial (0–6 s) and subsequent viewing periods.

Next, the stability of gaze structure was evaluated using HMM-based gaze-state sequence metrics. For the maximum state probability (MaxP), the median value during the period after 6 s was 0.406 in the high-evaluation group and 0.401 in the low-evaluation group, yielding a small difference in favor of the high-evaluation group (difference ≈ 0.006, *p* = 0.383, *r*(RB) = 0.145; Table 2). Similarly, no substantial group difference was observed for the entropy-based measure of state distribution (Hnorm), with median values of approximately 0.556 for the high-evaluation group and 0.552 for the low-evaluation group (difference ≈ 0.002, *p* = 0.814; Figure 5). The effective number of states (NeffNorm) also showed only minimal differences between groups, indicating limited overall compression of the state distribution (difference ≈ 0.001, *p* = 0.659; Figure 6).

**Table 2:** Saccade direction stabilization metrics during the initial viewing period.

Metric	nPaired	median HighArt	median LowArt	medianDiff_HminusL	p_signrank	r_rank Biserial
Hnorm	48	0.556	0.552	0.002	0.814	0.039
NeffNorm	48	0.284	0.282	0.001	0.659	0.073
MaxP	48	0.406	0.407	0.006	0.383	0.145
TV	48	0.672	0.674	−0.009	0.242	−0.194
Switch	48	0.875	0.853	−0.003	0.596	−0.093

Comparison of saccade direction bias metrics between high- and low-aesthetic-evaluation groups during the initial viewing period (0–3 s after stimulus onset). For each artwork and participant (*n* = 48 paired observations), saccades were classified by movement direction, and five directional bias indices (Hnorm, NeffNorm, MaxP, TV, and Switch) were computed. The table reports the median values for the high- and low-evaluation conditions, their median differences (High minus Low), Wilcoxon signed-rank test p-values, and rank-biserial correlation coefficients. No significant group differences were observed for any of the directional bias measures, indicating that early saccade directionality did not systematically differ as a function of aesthetic evaluation.

**Figure 7:**
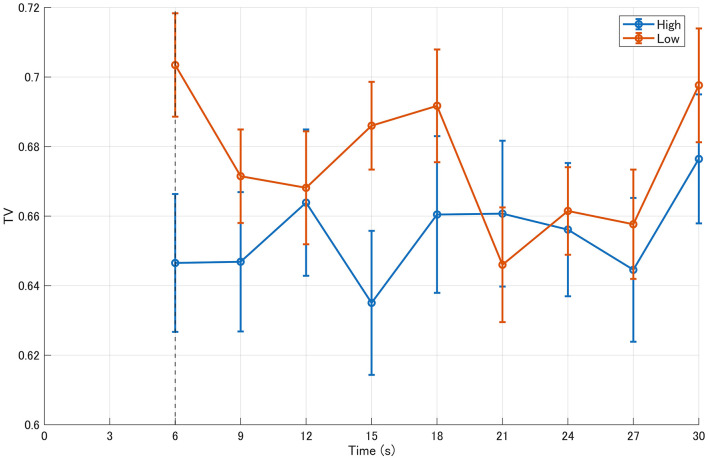
Time-course of transition variability (TV, BIN) for the 12-image and 20-image stimulus sets. Time-course of transition variability (TV), reflecting variability in gaze state transitions, computed in 3-s bins for high- and low-aesthetic-evaluation groups. Results are presented for the 12-image and 20-image stimulus sets. Median values and interquartile ranges are shown. The dashed vertical line marks the end of the initial viewing period (6 s).

**Figure 8:**
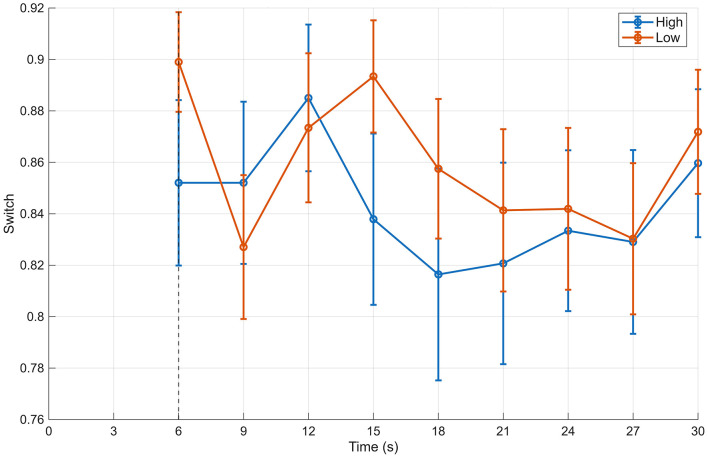
Time-course of state switching probability (Switch, BIN) for the 12-image and 20-image stimulus sets. Time-course of state switching probability (Switch), indicating the likelihood of transitions between gaze states, computed in 3-s bins for high- and low-aesthetic-evaluation groups. Data are shown separately for the 12-image primary stimulus set and the 20-image secondary stimulus set. Points indicate medians and error bars denote interquartile ranges. The vertical dashed line represents the end of the initial viewing phase (6 s).

In contrast, when focusing on transition variability—a measure reflecting the dispersion of state transitions—the median value was approximately 0.672 in the high-evaluation group and 0.674 in the low-evaluation group, indicating slightly lower variability in the high-evaluation group (difference ≈ −0.009, *p* = 0.242, *r*(RB) = −0.194). Although this effect did not reach statistical significance, the pattern suggests a tendency for gaze transitions in the high-evaluation group to converge toward a more stable structure after 6 s (Figures 7, 8).

Taken together, these results indicate that during the mid-viewing phase, gaze-state sequences in the high-evaluation group did not undergo abrupt changes in any single metric. Instead, the overall gaze structure became organized through a combination of increased cumulative dwell time around depth points and gradual stabilization of state transitions. In contrast, the low-evaluation group exhibited relatively sustained exploratory gaze behavior, and a comparable degree of structural convergence was not clearly observed. Thus, the temporal divergence identified in Section 4.1 subsequently manifested as stabilization and cumulative differentiation of gaze structure during the later stages of viewing.

### 5.3 Group differences in depth localization measures

Using response data from the Depth Localization Task for the primary stimulus set, depth-related measures were computed for the most frequently selected depth point for each artwork and compared between the high- and low-evaluation groups. Specifically, subjective perceived depth distance (*Distance_km*), time to first recognition of the depth point (*Depth recognition completion time*), and time required to estimate depth distance (*Calculate distance time*) were analyzed.

The results showed that subjective depth distance (*Distance_km*) was significantly larger in the high-evaluation group (*n* = 126, *M* = 46.6) than in the low-evaluation group (*n* = 112, *M* = 15.6), *t*_(203.6)_ = 4.89, *p* = 2.08 × 10^−6^, Cohen's *d* = 0.62 [95% CI: 0.36–0.88]. In addition, the time to recognize the depth point (*Depth recognition completion time*), expressed as within-participant normalized values, was significantly shorter in the high-evaluation group (*M* = −0.27) than in the low-evaluation group (*M* = 0.09), *t*_(223.4)_ = −3.13, *p* = 0.002, *d* = −0.41 [95% CI: −0.67 to −0.15]. In contrast, no significant group difference was observed for the time required to compute depth distance (*Calculate distance time*), *t*_(228.1)_ = 1.65, *p* = 0.10, *d* = 0.21.

These findings indicate that participants in the high-evaluation group perceived depth points as being located at greater distances and recognized these locations more rapidly, whereas the processing time required to compute the distance itself did not differ between groups. Thus, group differences were not associated with the initial arrival of gaze at depth points, but rather with the subsequent processes of interpreting and confirming depth. This pattern is consistent with the temporal divergence observed in gaze behavior during free viewing.

### 5.4 Replication in the secondary stimulus set

To examine the generality of the temporal divergence observed in the primary stimulus set, the same analytical procedures were applied to a secondary stimulus set consisting of 20 landscape prints by *Ukiyo-e* artists active during the same historical period as Hokusai and Hiroshige (secondary stimulus set; *n* = 45, 20 artworks). Detailed temporal profiles of gaze metrics for this stimulus set are presented in Supplementary Figures 3-1–3-3.

First, effect-size trajectories based on cumulative (0–secN) measures were examined. During the initial exploratory phase (0–6 s), group differences were small across all metrics, replicating the pattern observed in the primary stimulus set (*n* = 48, 12 artworks). For example, the effect size (r) for cumulative DP dwell time (Dwell_ms) was approximately −0.04 at 3 s and −0.06 at 9 s.

During the mid-to-late viewing phase (after 6 s), group differences in the secondary stimulus set were maintained in the negative direction, with effect sizes of approximately *r* ≈ −0.05 at 15 s and *r* ≈ −0.07 at 30 s, indicating a relative increase in dwell time around depth points in the high-evaluation group. However, the magnitude of this amplification was limited compared with that observed in the primary stimulus set, in which a monotonic increase in effect size over time was evident (reaching *r* ≈ −0.28 at 30 s). This difference is also apparent in the cumulative trajectories shown in Supplementary Figures 3-1, 3-2.

A similar pattern was observed for the cumulative hit-count measure. In the secondary stimulus set, effect sizes remained within a narrow range of approximately *r* ≈ −0.04 to −0.07 across all time windows, and the pronounced mid-to-late amplification observed in the primary stimulus set was not reproduced. Nevertheless, the direction of the group difference was consistent with the main analysis, indicating that DP-related fixations tended to be relatively stronger in the high-evaluation group.

In contrast, the time to first fixation on depth points (FirstHit_ms) showed only small effect sizes in the secondary stimulus set (*r* ≈ 0.05–0.09), with no consistent temporal trend. FirstHit_ms reflects a low-level index of attentional allocation to potential depth cues, suggesting that the speed with which gaze initially reached depth candidates was comparable across evaluation groups. Importantly, this measure reflects a different processing stage from the depth-recognition completion time measured in the Depth Localization Task and does not preclude the possibility of group differences emerging during later stages of cognitive integration and confidence formation following initial access.

Taken together, these results indicate that in the secondary stimulus set, group differences were minimal during the initial exploratory phase and a temporal divergence in gaze metrics emerged during the mid-viewing phase, replicating the overall temporal pattern observed in the primary stimulus set. However, the magnitude of amplification and cumulative differentiation was more limited. These findings suggest that gaze-structure divergence associated with EDEN is modulated by stimulus content and compositional characteristics, rather than being uniformly expressed across all artworks.

### 5.5 Results summary

Across analyses, no pronounced group differences were observed during the initial exploratory phase (0–6 s), whereas a stable divergence in gaze behavior emerged during the mid-viewing phase. In the high-evaluation group, fixation frequency and dwell time around compositional depth points increased and remained stable over time, while the low-evaluation group exhibited more sustained exploratory viewing. These temporal patterns were robust across the primary stimulus set and qualitatively replicated in the secondary stimulus set, albeit with reduced magnitude.

Furthermore, analyses based on HMM-derived metrics indicated that, in the high-evaluation group, increased concentration of gaze-state distributions (higher MaxP), reduced uncertainty of state sequences (lower entropy), and stabilization of transition structure (lower transition variability) emerged concurrently during the mid-viewing phase. Together, these patterns suggest a process in which gaze behavior becomes organized into stable state sequences centered on specific compositional elements.

These temporal divergence and amplification effects were consistently observed across the twelve artworks comprising the primary stimulus set. In the secondary stimulus set (20 artworks), the magnitude of the effects was attenuated; however, the overall direction of the effects was preserved, indicating a qualitatively similar temporal pattern. Exploratory analyses of directional gaze features revealed no significant group differences (see Supplementary material).

## 6 General discussion

While few group differences were observed during the initial exploratory phase immediately after viewing onset, a consistent temporal pattern was observed in which the organization of gaze behavior diverged during the mid-viewing phase and the resulting differences were maintained over time. These results indicate that the structuring of gaze behavior related to compositional depth cues does not occur uniformly throughout viewing, nor is it necessarily expressed at the earliest stage.

In the present study, this pattern is described using the concept of Early Depth Engagement (EDEN), which refers to the temporal emergence, divergence, and maintenance of depth-related gaze organization during free viewing. Importantly, EDEN is not intended to denote a specific cognitive mechanism or evaluative process, but rather to provide a descriptive framework for organizing observed temporal patterns in gaze behavior.

### 6.1 Temporal characteristics of depth-related gaze divergence

The results showed that during the initial exploratory phase, gaze behavior in both evaluation groups was predominantly dispersed, and no stable group differences were observed in measures related to depth points. By contrast, from approximately 6 s after viewing onset, gaze behavior began to diverge between groups. In the high-evaluation group, gaze increasingly concentrated on regions containing compositional depth points, and this tendency was maintained during subsequent viewing. In the low-evaluation group, exploratory gaze behavior persisted to a greater extent, and comparable stabilization was less pronounced. These findings suggest that depth-related differences in gaze behavior during free viewing emerge at an intermediate stage rather than immediately upon stimulus presentation. This temporal profile differs from effects reported in tasks requiring rapid judgment or explicit decision-making, and instead reflects changes unfolding as viewing progresses under unconstrained conditions. Differences between gaze-based measures and task-based depth localization measures can be understood in terms of the stage of processing they reflect. Gaze measures capture the temporal dynamics of visual exploration during free viewing, whereas task-based measures reflect outcomes following explicit depth interpretation. Accordingly, the two sets of measures index different aspects of depth-related processing and should be regarded as complementary rather than redundant.

### 6.2 Cumulative organization of gaze behavior

The observed divergence in gaze behavior was not transient but was maintained throughout the mid- to late-viewing phases. In the high-evaluation group, cumulative dwell time around depth-related regions increased and remained stable over time, whereas the low-evaluation group showed weaker accumulation. In addition, measures derived from gaze-state sequences indicated tendencies toward reduced transition variability in the high-evaluation group, suggesting gradual organization of gaze structure. These patterns indicate that depth-related gaze engagement during free viewing can accumulate over time, resulting in increasingly differentiated viewing behavior between evaluation groups. However, these changes were expressed gradually and across multiple measures, rather than as abrupt shifts in any single index.

### 6.3 Differences across stimulus sets

The temporal pattern of divergence observed in the primary stimulus set was also present in the secondary stimulus set, although the magnitude of amplification was reduced. This suggests that the timing of divergence is not specific to a particular subset of artworks, while the strength of cumulative differentiation may depend on compositional characteristics of the stimuli. Importantly, the reduced magnitude observed in the secondary set does not indicate the absence of the phenomenon, but rather suggests that the expression of depth-related gaze organization may vary with stimulus content and artistic style.

### 6.4 Free viewing and aesthetic engagement

A key feature of the present study is that all analyses were conducted under free viewing conditions without explicit evaluative demands. Despite this, systematic differences in gaze organization related to aesthetic evaluation were observed. This indicates that differences in aesthetic evaluation can be associated with differences in perceptual exploration, even in the absence of explicit judgment during viewing. From this perspective, EDEN describes a stage of perceptual organization that precedes explicit interpretation or evaluative reporting. While the present results do not imply that depth-related gaze organization directly determines aesthetic judgment, they suggest that such organization forms part of the perceptual context within which evaluation later occurs. **EDEN can thus be positioned as a perceptual foundation that precedes RAIC (Reading an Artist's Intention from the Composition), providing the temporally organized gaze structure upon which subsequent interpretative processes are built**.

### 6.5 Limitations and future directions

Several limitations should be noted. First, the magnitude of depth-related gaze divergence varied across stimulus sets, indicating sensitivity to compositional properties. Second, the present study focused exclusively on eye-movement behavior and did not directly measure neural or physiological processes underlying depth perception or aesthetic engagement. Third, only free viewing was examined; how these temporal patterns may change under explicit judgment or time-constrained conditions remains to be investigated. Future studies combining eye tracking with neural or physiological measures, as well as systematic manipulation of task demands, will be necessary to further clarify the scope and functional significance of depth-related gaze organization during art viewing.

## 7 Conclusion

The present study examined gaze behavior during free viewing from a temporal-dynamics perspective and demonstrated that engagement with compositional depth cues leads to divergence and stabilization of gaze structure at an intermediate stage of the viewing process. The transition observed at approximately 6 s after viewing onset reflects a shift from exploratory to structured viewing, rather than an effect of immediate fixation or momentary judgment.

By conceptualizing this temporal transition as Early Depth Engagement (EDEN), the present study reframes depth perception not as a static compositional feature, but as a dynamic perceptual process that emerges and becomes organized over time during viewing. EDEN provides a framework for capturing how gaze behavior incorporates depth cues as structural anchors, supporting the internal reconstruction of spatial organization from two-dimensional visual input.

Together, these findings support a view of aesthetic experience as a temporally unfolding perceptual and exploratory process, rather than as the outcome of a single evaluative moment, and offer new insights into the temporal foundations of compositional understanding and aesthetic engagement.

## Data Availability

All data and materials (i.e., fixation data, pupil size, impression evaluation value, analysis code) are publicly available via the Open Science Framework and can be accessed at https://osf.io/ghv87?view_only=85de84e5d63948ab92001d9deebdb2f1/.
