# Supplementary material for: Early Depth Engagement in Art Perception: visual dynamics and aesthetic experience
Source: Front Psychol. 2026 Apr 16;17:1781822. doi: 10.3389/fpsyg.2026.1781822 (PMC13130483; doi:10.3389/fpsyg.2026.1781822)

Supplementary Figure 1.

List of 20 digital images provided free of charge, including the artist, title, and estimated year of production.

Images courtesy of the British Museum, London; the Honolulu Museum of Art; and the Chazen Museum of Art, University of Wisconsin–Madison.

Gakutei Harunobu

Aji River Ishibashi (Stone Bridge) at Tenpözan, Osaka c. 1818–1830

Image courtesy of Honolulu Museum of Art.

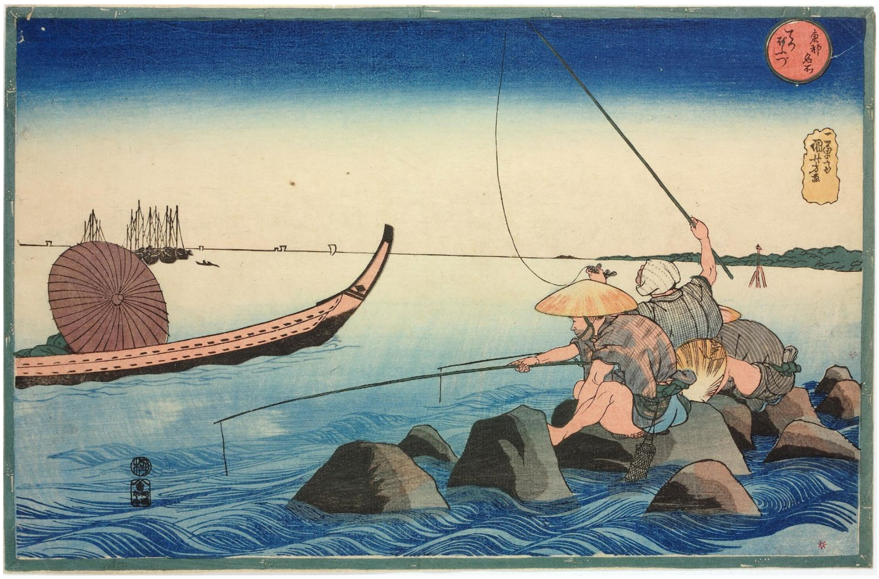

Shoutei Hokuju

Sea View from Benten Shrine at Susaki c.1818-1830

Image courtesy of Honolulu Museum of Art.

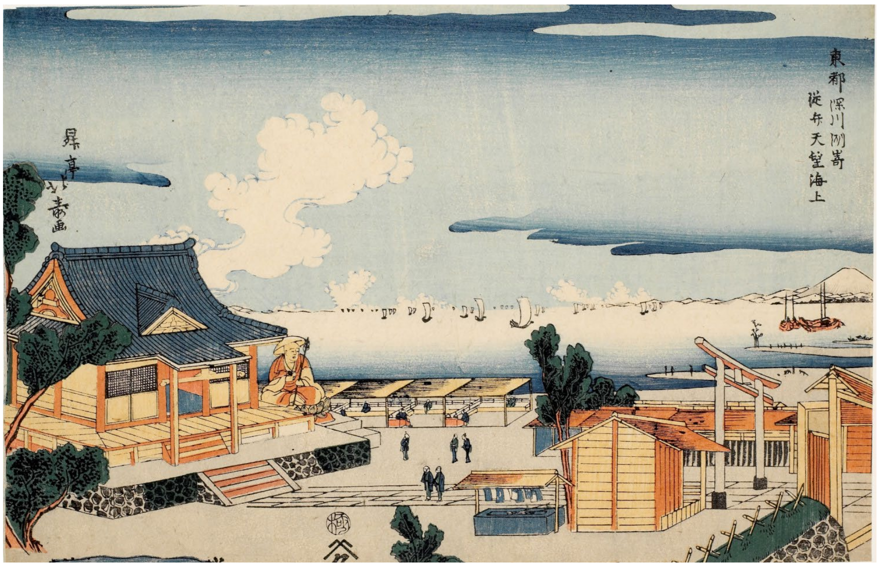

Gakutei Harunobu

Views of Tempözan c.1833

Image courtesy of Honolulu Museum of Art.

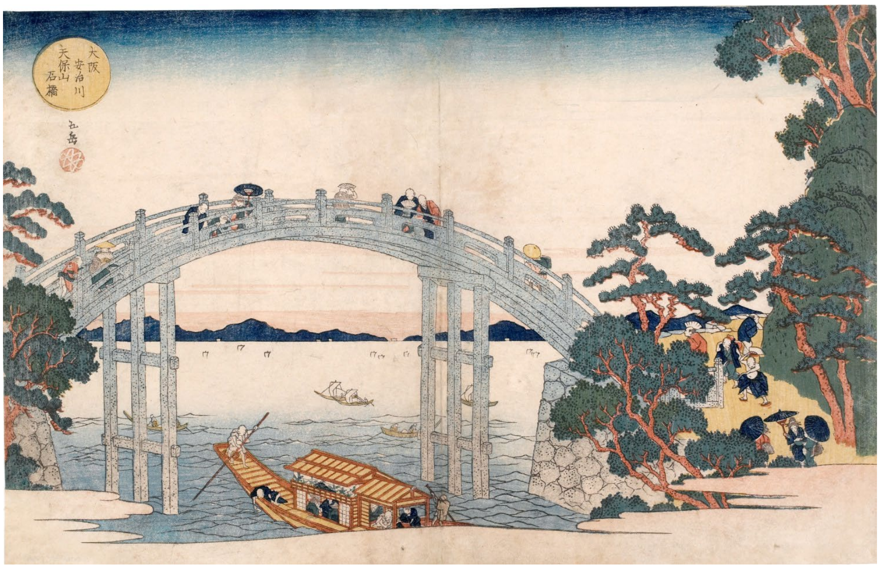

Utagawa Kuniyoshi

In the Snow at Tsukahara in Sado Province c.1831

Image courtesy of Honolulu Museum of Art.

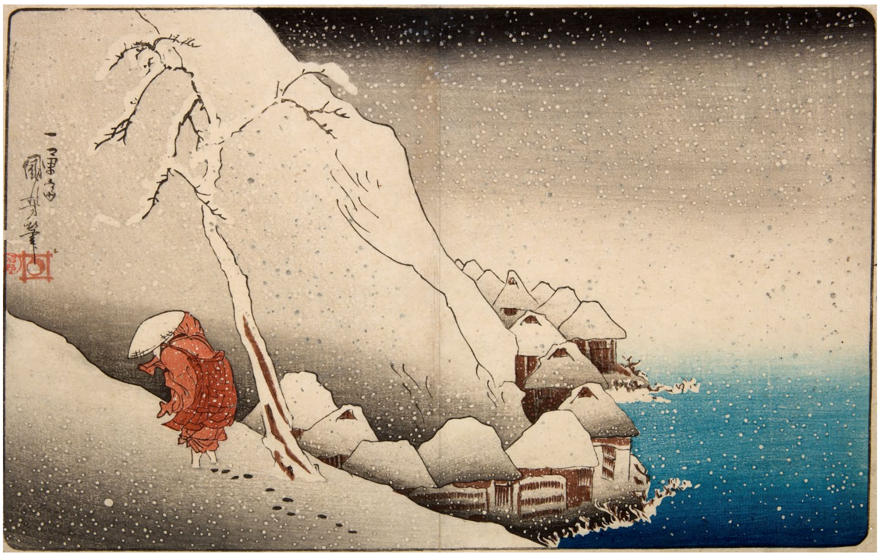

Utagawa Kuniyoshi

View of Tamura Ferry on the Road to Ōyama in Sagami Province c.1839

Image courtesy of Chazen Museum of Art, University of Wisconsin–Madison.

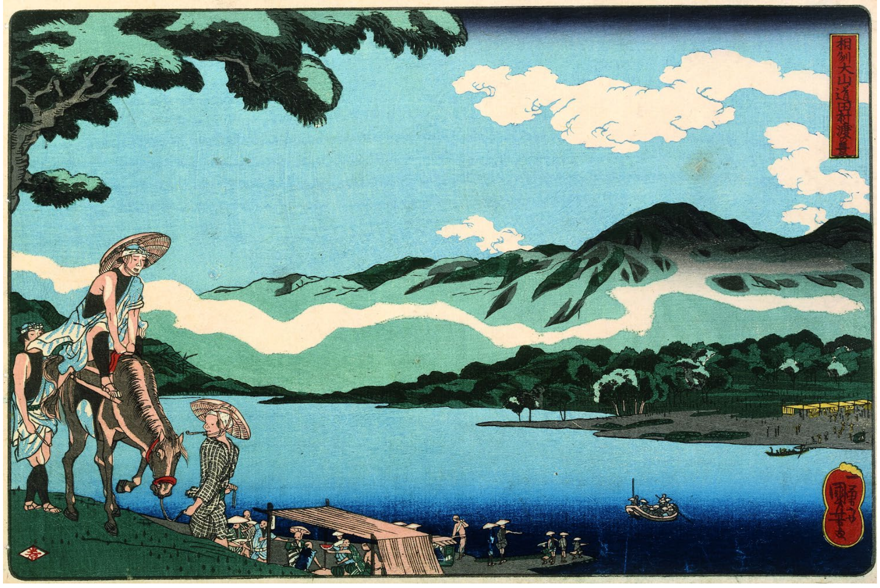

Utagawa Kuniyoshi

Picture of Miyatogawa c.1830-34

Image courtesy of Honolulu Museum of Art.

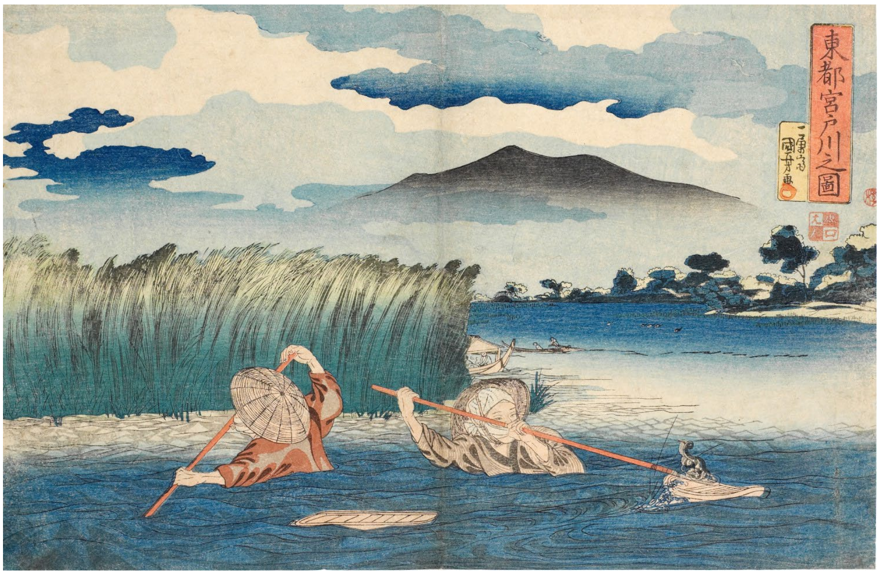

Keisai Eisen

Asakusa Kinryüzan Temple c.1830-44

Image courtesy of Honolulu Museum of Art.

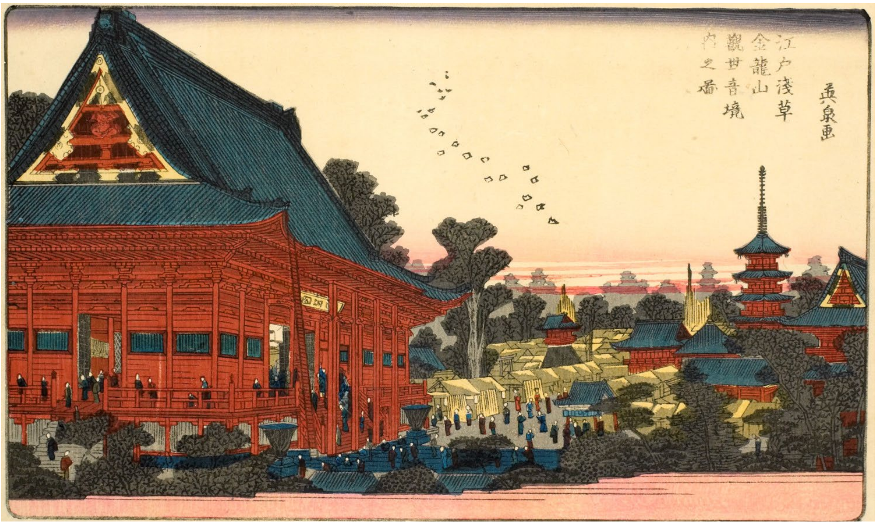

Keisai Eisen

Crouching dragon plum grove at Umeyashiki, Edo c.1835

Image courtesy of Honolulu Museum of Art.

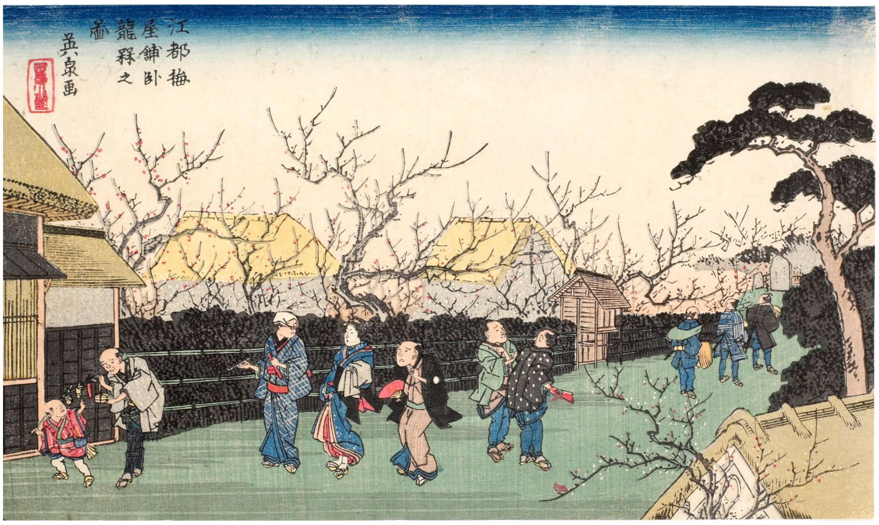

Keisai Eisen

Itahana c.1830-44

Image courtesy of Minneapolis Institute of Art

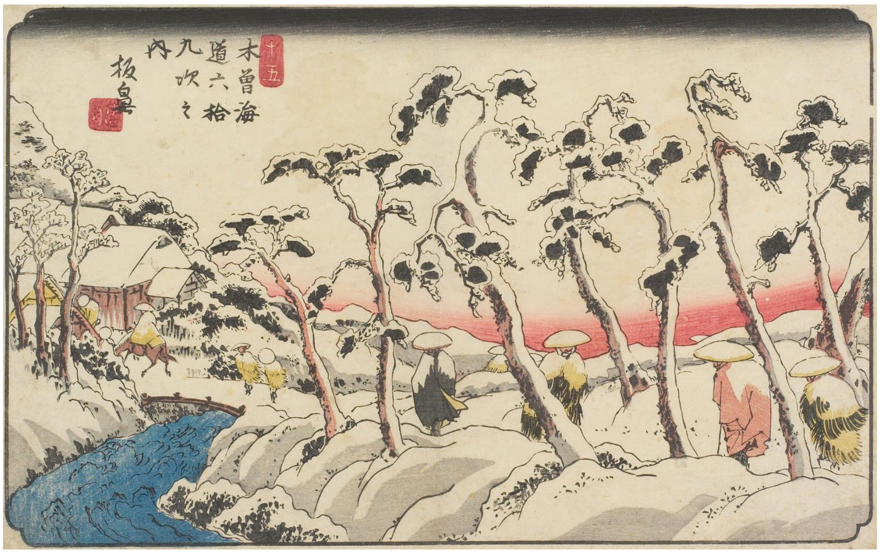

Keisai Eisen

View of Hatcho Embankment at Kumagaya Station, no. 9 in the series The Sixty-nine Stations of the Kisokaido c.1835

Image courtesy of Chazen Museum of Art, University of Wisconsin–Madison.

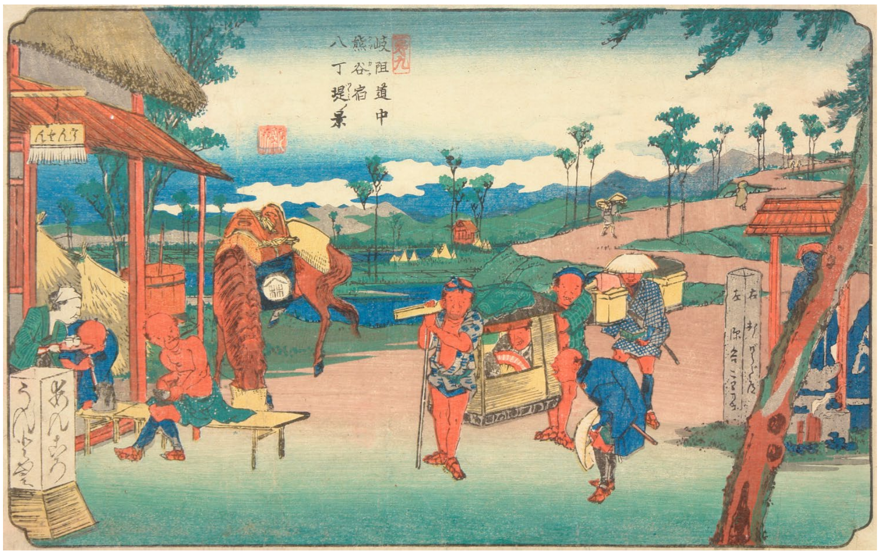

Keisai Eisen

Picture of the Theater District in Edo During the Kaomise Season, from a series of Famous Places in Edo c.1841

Image courtesy of Chazen Museum of Art, University of Wisconsin–Madison.

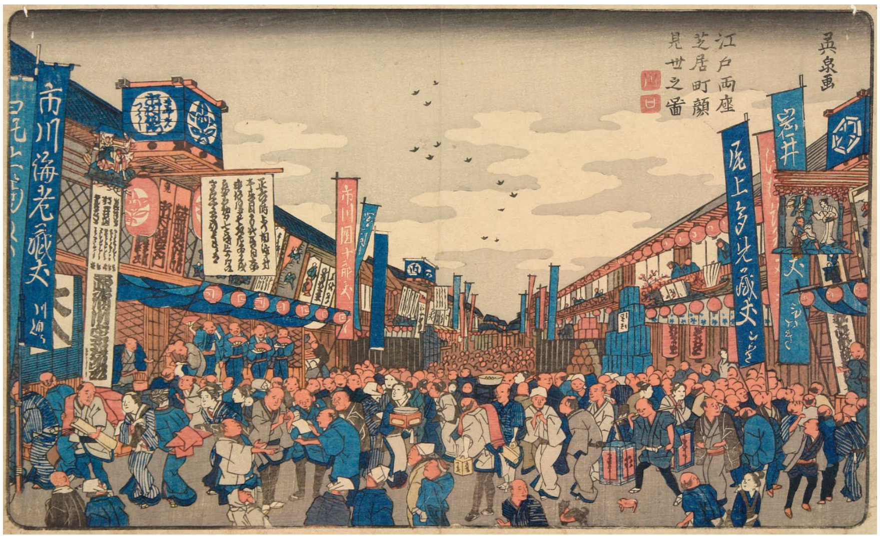

Utagawa Kuniyoshi

A Picture of Mitsumata in the Eastern Capital, from a series of Views of the Eastern Capital c.1835

Image courtesy of Chazen Museum of Art, University of Wisconsin–Madison.

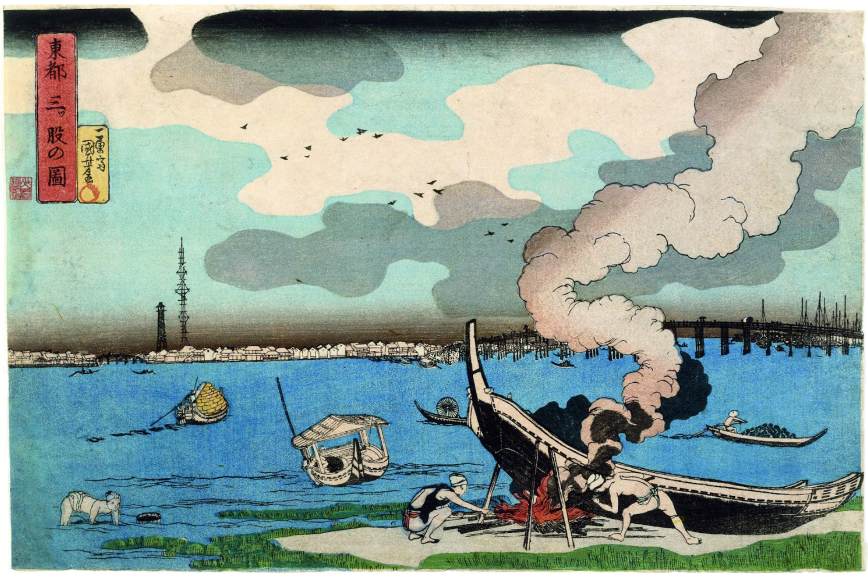

No. 4, Urawa Station: Distant View of Mount Asama ,
from the series The Sixty-nine Stations of the Kisokaidô Road c.1835-38
Image courtesy of Chazen Museum of Art, University of
Wisconsin–Madison.

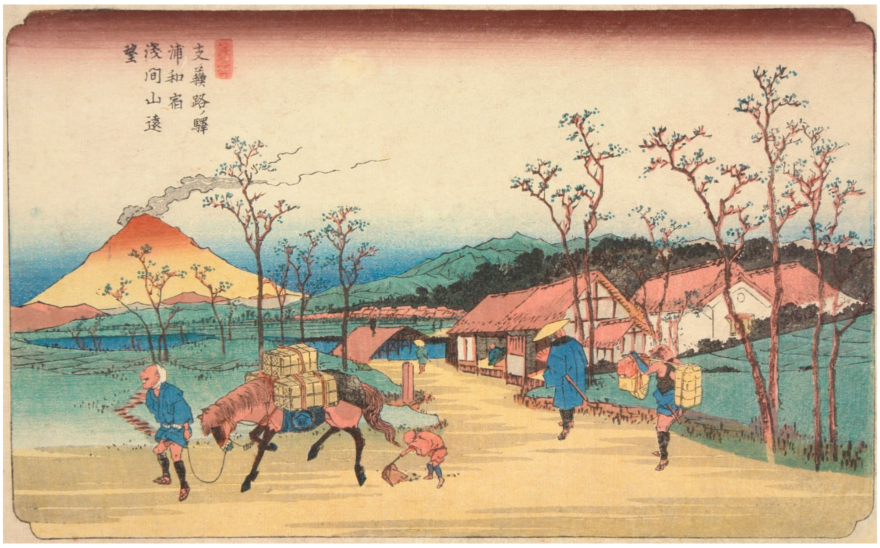

Keisai Eisen
Warabi no Eki: Toda-gawa Watashi / Kisokaidō”（「木曽街道 蕨之駅
戸田川渡場」） c. 1835-1842
Image courtesy of Chazen Museum of Art, University of
Wisconsin–Madison.

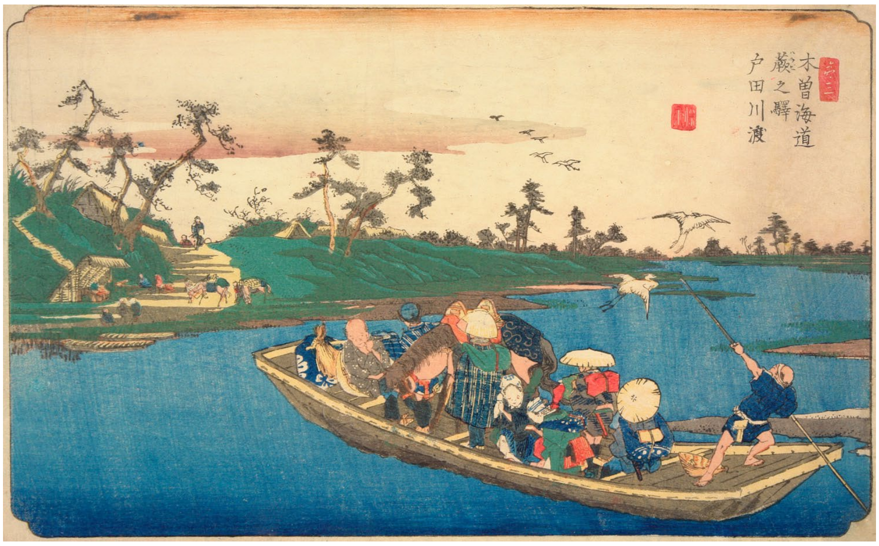

Keisai Eisen

Distant View of Mt. Fuji from Fukiage, no. 8 from the series

The Sixty-nine Stations of the Kisokaido c.1835

Image courtesy of Chazen Museum of Art, University of Wisconsin–Madison.

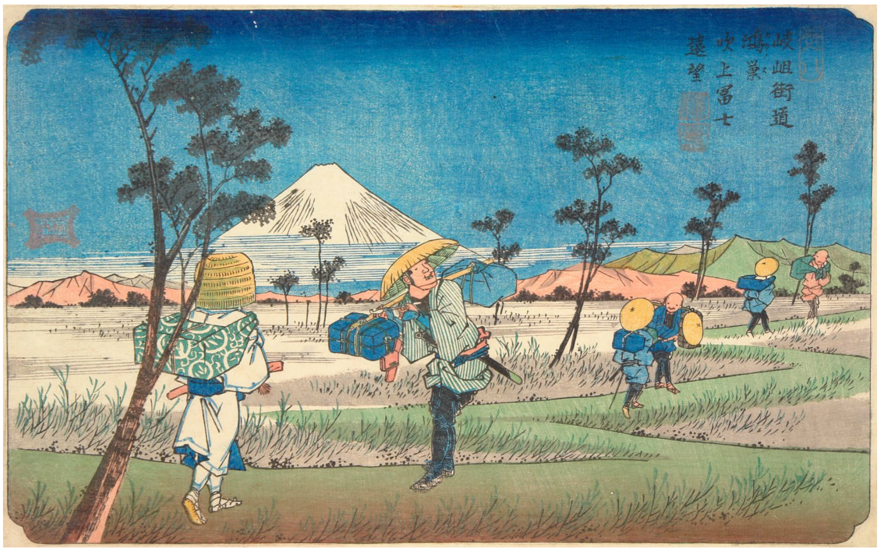

Utagawa Kuniyoshi

Famous Places in the Eastern Capitol Fishermen at Teppozu c.1830

Image courtesy of the British Museum, London.

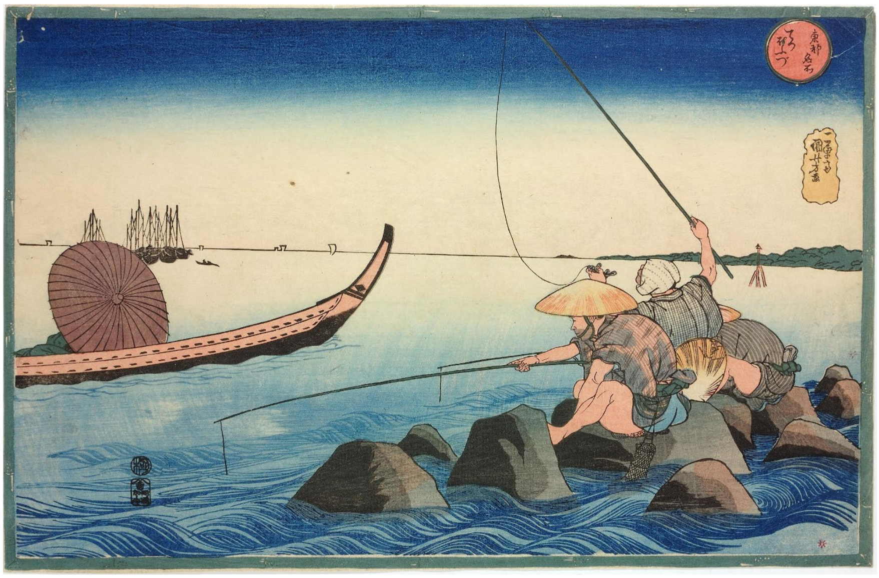

Utagawa Kuniyoshi

Kasumigaseki, from the series Famous Places in the Eastern Capital (Tôto meisho) c.1830-35

Image courtesy of Honolulu Museum of Art.

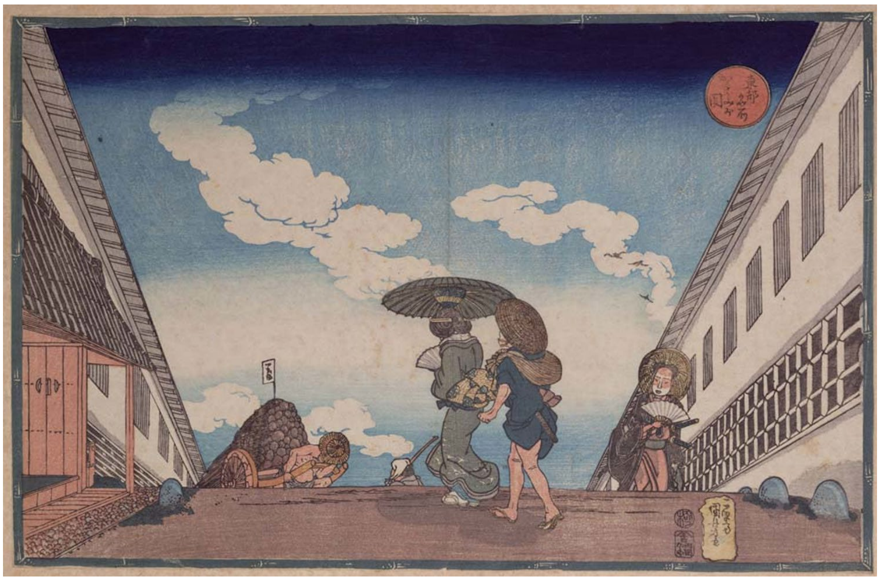

Keisai Eisen

Shinobazu Pond C. 1830s.

Image courtesy of the British Museum, London.

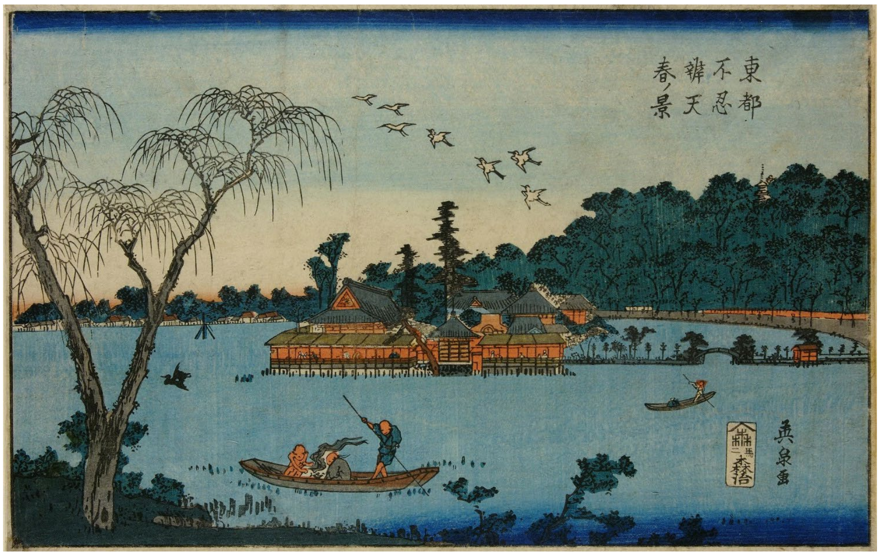

Keisai Eisen

Omiya yado Fuji enkei (no.50) / Kisokaido c.1835-1842

Image courtesy of the British Museum, London.

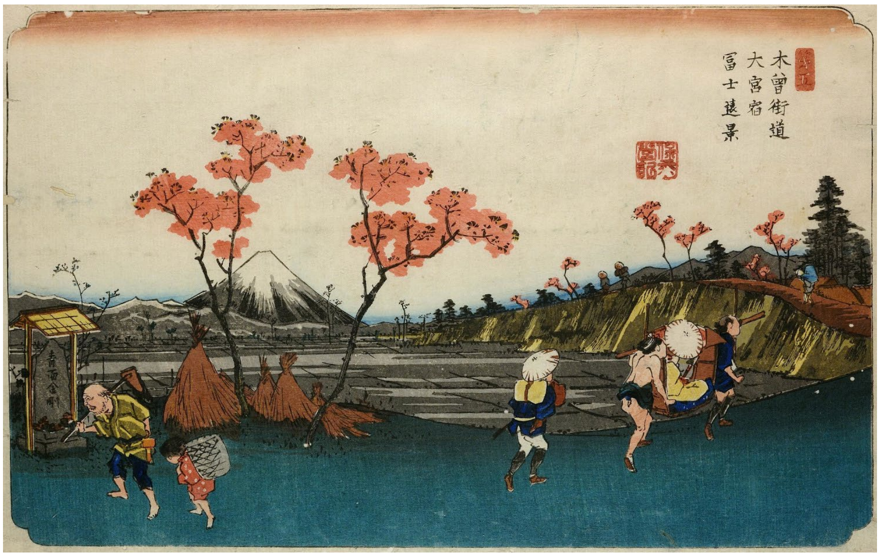

Keisai Eisen

The Ferry on the Kanna River at Honjo Station, no. 11 from the series The Sixty-nine Stations of the Kisokaido c. 1835

Image courtesy of Chazen Museum of Art, University of Wisconsin–Madison.

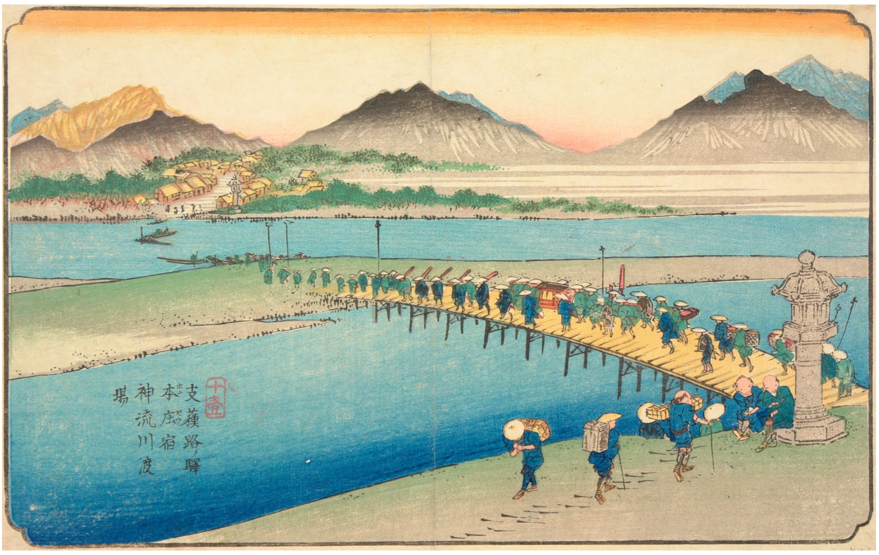

Supplement: Supplementary file 1 [file Data_Sheet_1.pdf]
