# Supplementary material for: Early Depth Engagement in Art Perception: visual dynamics and aesthetic experience
Source: Front Psychol. 2026 Apr 16;17:1781822. doi: 10.3389/fpsyg.2026.1781822 (PMC13130483; doi:10.3389/fpsyg.2026.1781822)
Supplement: Supplementary file 2 [file Data_Sheet_2.pdf]

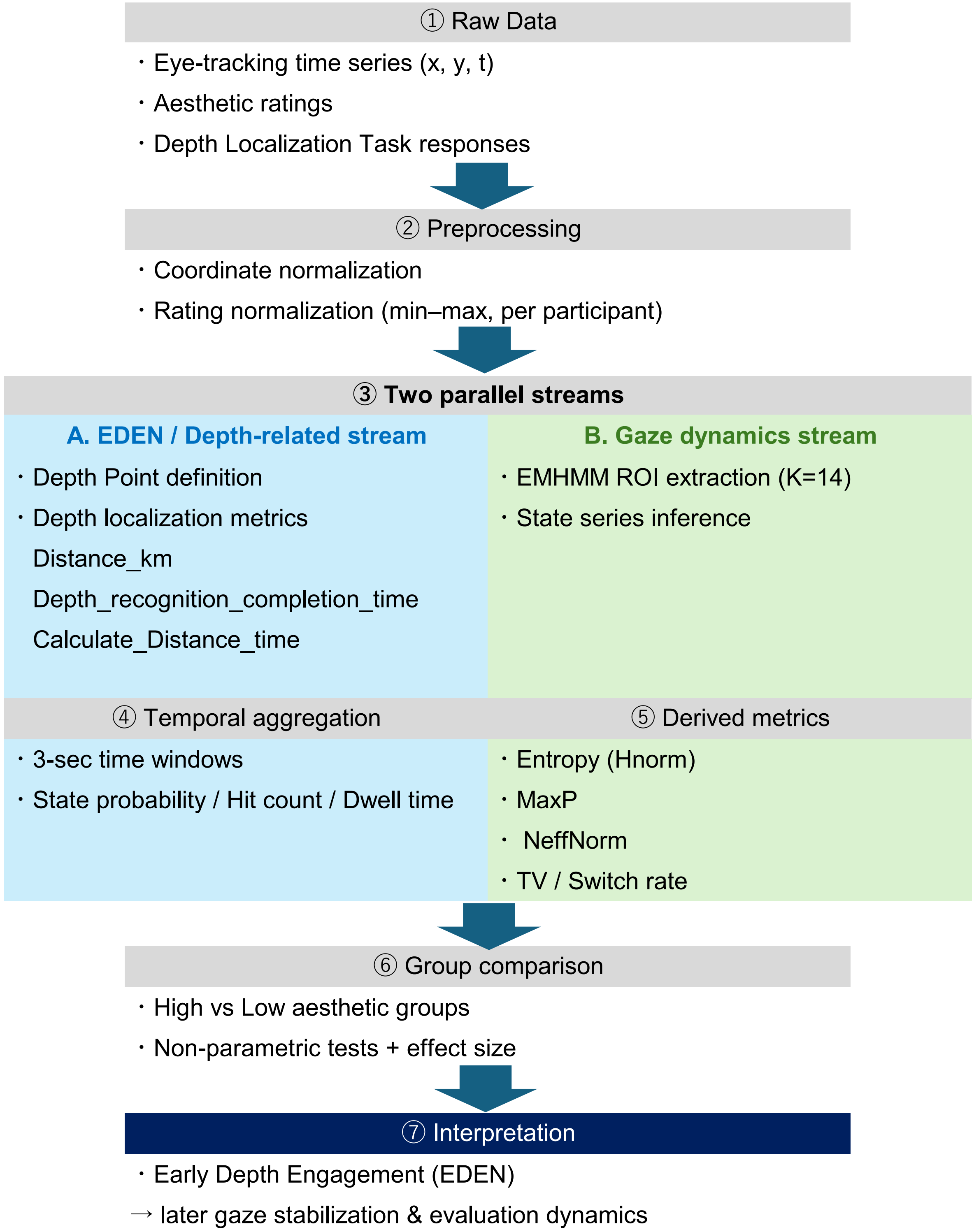

Supplementary Figure 2.
Analysis pipeline for saccade direction bias during the initial viewing period. Saccades occurring within the first 0–3 s after stimulus onset were extracted from the gaze time series and classified according to their movement direction (horizontal, vertical, or oblique). For each participant and stimulus, directional distributions were computed, and a direction bias index was derived to quantify deviations from an isotropic distribution. These indices were compared between the high- and low-aesthetic-evaluation groups to examine whether early exploratory eye movements exhibited systematic directional preferences related to aesthetic evaluation. No significant group differences were observed in these directional measures.
