# Supplementary figures and images for: Early Depth Engagement in Art Perception: visual dynamics and aesthetic experience
Source: Front Psychol. 2026 Apr 16;17:1781822. doi: 10.3389/fpsyg.2026.1781822 (PMC13130483; doi:10.3389/fpsyg.2026.1781822)

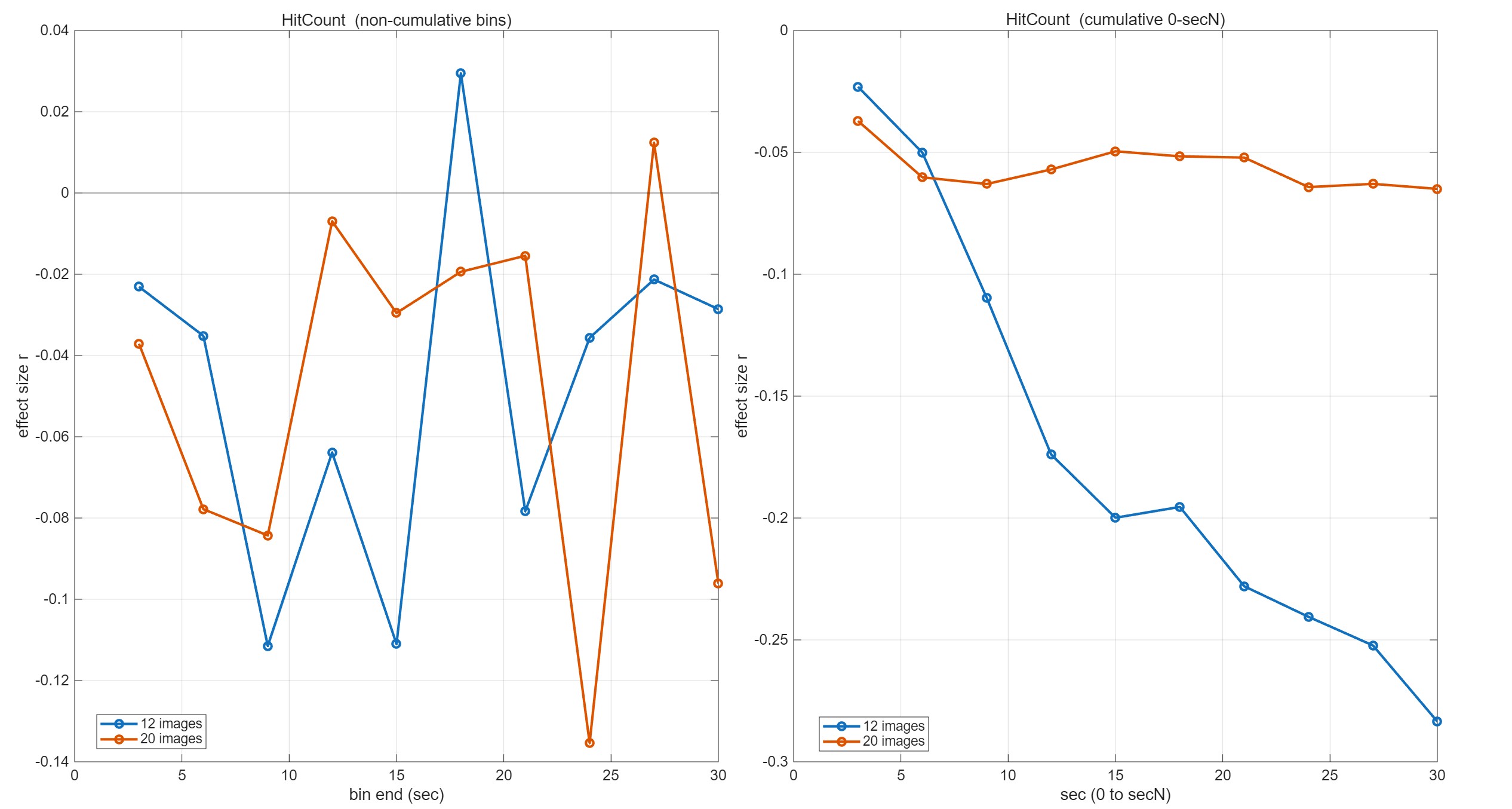

Supplement: Supplementary file 4 [file Image_1.jpeg]

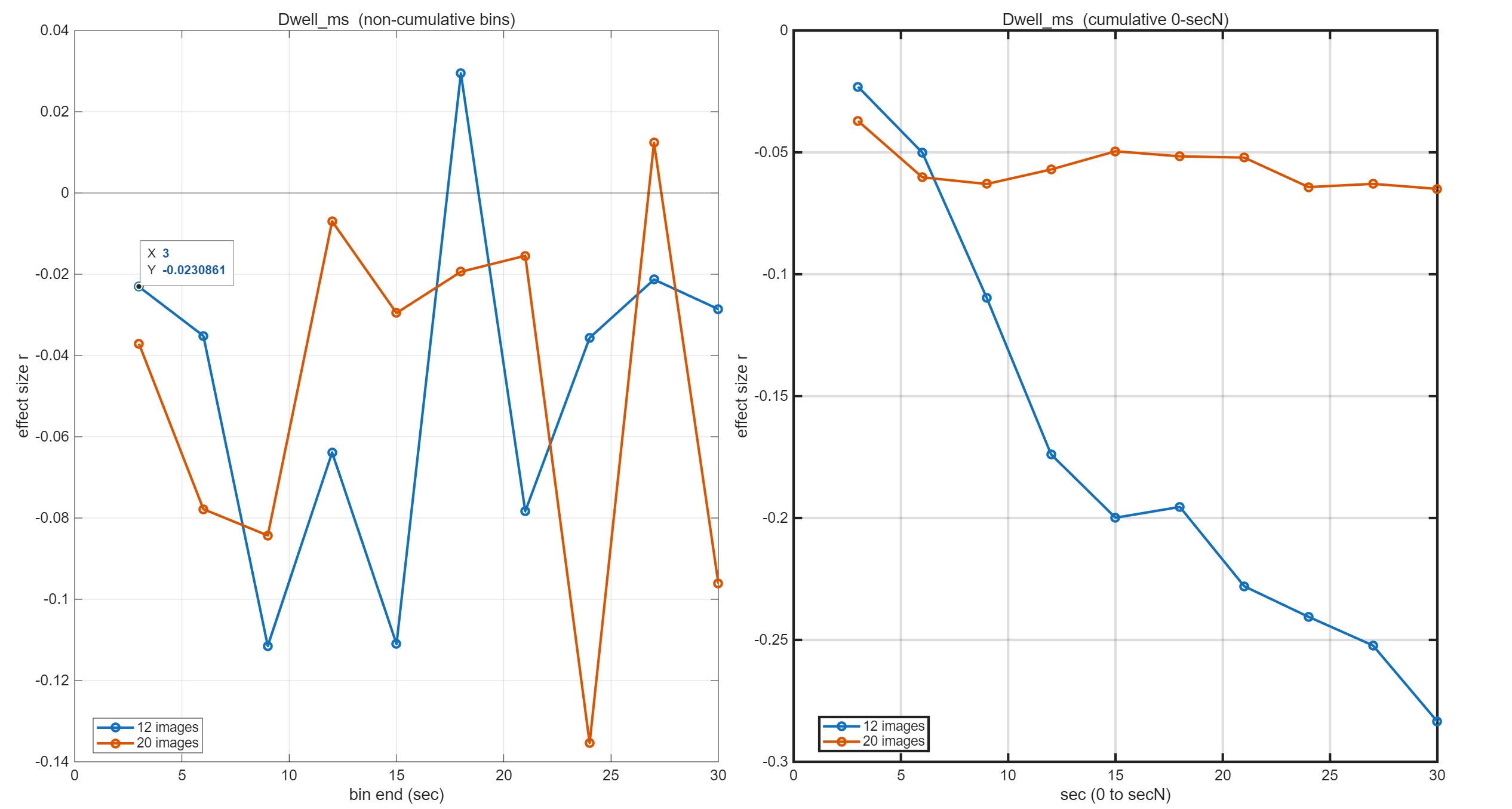

Supplement: Supplementary file 5 [file Image_2.jpeg]

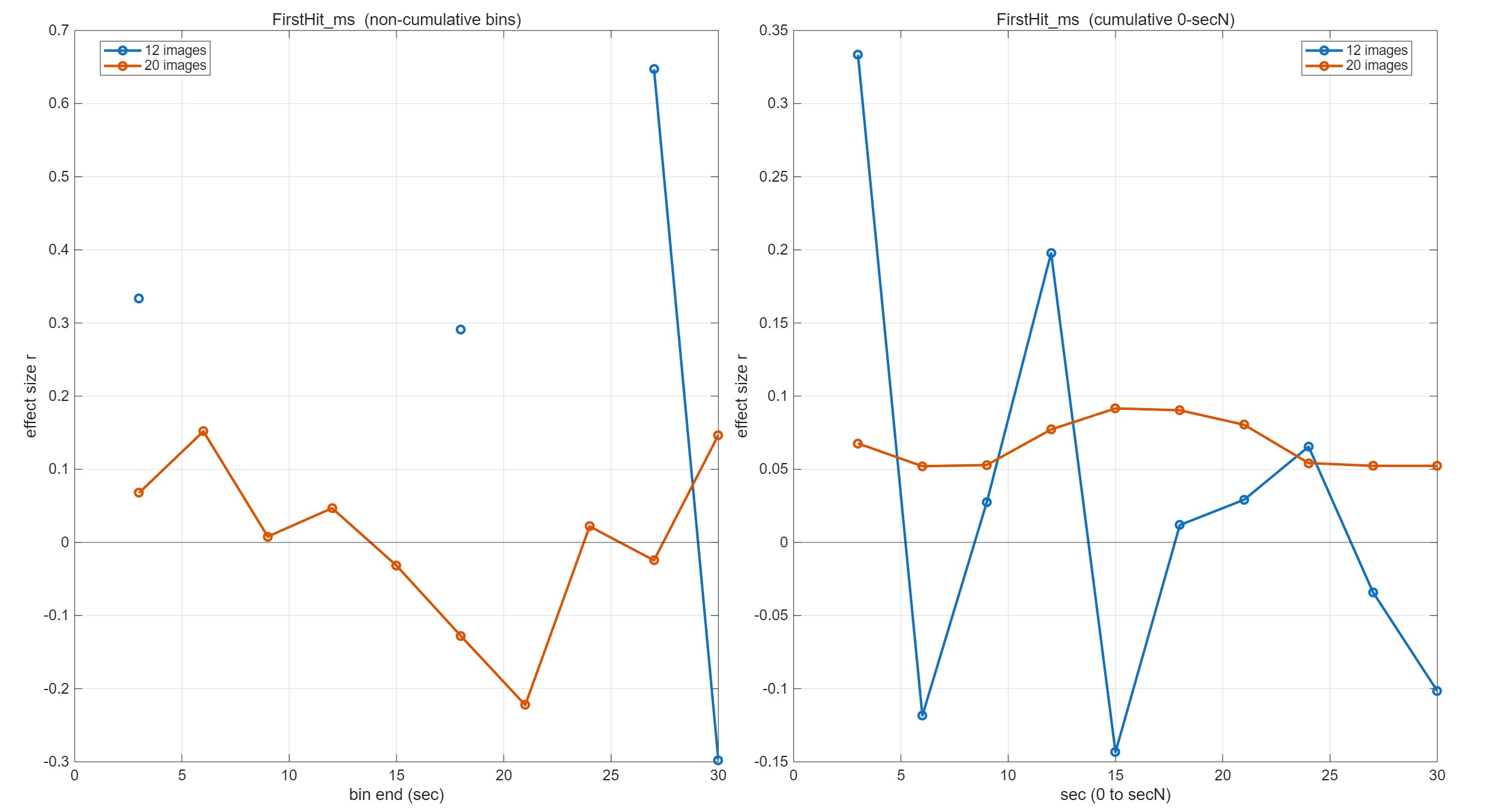

Supplement: Supplementary file 6 [file Image_3.jpeg]
